# Luteolin and abyssinone II as potential inhibitors of SARS-CoV-2: an in silico molecular modeling approach in battling the COVID-19 outbreak

**DOI:** 10.1186/s42269-020-00479-6

**Published:** 2021-01-20

**Authors:** Mohammad Mahfuz Ali Khan Shawan, Sajal Kumar Halder, Md. Ashraful Hasan

**Affiliations:** Department of Biochemistry and Molecular Biology, Jahangirnagar University, Savar, Dhaka, 1342 Bangladesh

**Keywords:** Abyssinone II, COVID-19 pandemic, Flavonoids, Luteolin, Molecular docking, Molecular dynamics simulation, SARS-CoV-2

## Abstract

**Background:**

At present, the entire world is in a war against COVID-19 pandemic which has gradually led us toward a more compromised “new normal” life. SARS-CoV-2, the pathogenic microorganism liable for the recent COVID-19 outbreak, is extremely contagious in nature resulting in an unusual number of infections and death globally. The lack of clinically proven therapeutic intervention for COVID-19 has dragged the world’s healthcare system into the biggest challenge. Therefore, development of an efficient treatment scheme is now in great demand. Screening of different biologically active plant-based natural compounds could be a useful strategy for combating this pandemic. In the present research, a collection of 43 flavonoids of 7 different classes with previously recorded antiviral activity was evaluated via computational and bioinformatics tools for their impeding capacity against SARS-CoV-2. In silico drug likeness, pharmacophore and Absorption, Distribution, Metabolism, Excretion and Toxicity (ADMET) profile analysis of the finest ligands were carried out using DataWarrior, DruLiTo and admetSAR programs, respectively. Molecular docking was executed by AutoDock Vina, while molecular dynamics simulation of the target protein–ligand bound complexes was done using nanoscalable molecular dynamics and visual molecular dynamics software package. Finally, the molecular target analysis of the selected ligands within *Homo sapiens* was conducted with SwissTargetPredcition web server.

**Results:**

Out of the forty-three flavonoids, luteolin and abyssinone II were found to develop successful docked complex within the binding sites of target proteins in terms of lowest binding free energy and inhibition constant. The root mean square deviation and root mean square fluctuation values of the docked complex displayed stable interaction and efficient binding between the ligands and target proteins. Both of the flavonoids were found to be safe for human use and possessed good drug likeness properties and target accuracy.

**Conclusions:**

Conclusively, the current study proposes that luteolin and abyssinone II might act as potential therapeutic candidates for SARS-CoV-2 infection. In vivo and in vitro experiments, however, should be taken under consideration to determine the efficiency and to demonstrate the mechanism of action.

## Background

From December, 2019, the outset of a group of symptoms (i.e., dry cough, sore throat, fever, shortness of breath, lethargy, sputum production, headache, skin rash, loss of taste/smell along with diarrhea and vomiting in some cases) has begun to surge as a distinct type of pneumonia (acute respiratory infection of lungs) in Wuhan (Hubei Province), China (Guan et al. 2020). Later on, a newly discovered β-coronavirus was identified as the pathogenic organism causing the new type of pneumonia and the disease was named as coronavirus disease-2019 (COVID-19) (Guan et al. 2020). After immediate determination of the involvement of β-coronavirus in COVID-19, World Health Organization (WHO) named it as 2019-novel coronavirus (2019-nCoV) and due to the global health emergencies, International Committee of Coronavirus Study Group (ICCSG) proposed to use the name severe acute respiratory syndrome coronavirus 2 (SARS-CoV-2) for 2019-nCoV (Guo et al. 2020). Nowadays, COVID-19 has become a major public health concern because of the emergence of pandemic crisis around the world. In severe conditions, it may cause acute respiratory distress syndrome (ARDS) which is a different form of respiratory failure associated with inflammation of the lungs and characterized by the development of pulmonary infiltrate within and around the lungs resulting in septic shock and starvation of different organs for oxygen. At this moment, WHO declares COVID-19 outbreak as a public health threat of international concern because of its rapid dissemination and increased reproduction/transmission number (R0) day by day. As of November 13, 2020, it has already been transmitted to 220 different countries around the globe with 52,177,708 confirmed cases and 1,286,063 confirmed deaths (https://www.who.int/emergencies/diseases/novel-coronavirus-2019).

Coronaviruses are single-stranded RNA (+ sense) genome containing enveloped viruses belonging to Coronaviridae family and enter into the host cell by binding their spike proteins (S protein) with ACE-2 receptors on the cell surface of the host (Kuba et al. 2005). Inside the host cell, translated polyproteins from the viral RNA genome are processed to form mature/functional proteins, i.e., RNA-dependent RNA polymerase (RdRp), exoribonuclease and endoribonuclease using Mpro or 3CLpro with the assistance of PLpro (Hilgenfeld 2014). Hence, inhibition of the enzymatic activity of Mpro/3CLpro and PLpro would prevent viral replication, while blockage of the attachment of virus S protein with ACE-2 could cease the viral entry into the host cells (Towler et al. 2004; Du et al. 2009). Thus, due to their fundamental role in viral transmission, replication and pathogenesis, the above-mentioned virus and/or host components could be used as ideal molecular targets for developing novel and effective drug candidates against COVID-19.

Rapid transmission of COVID-19 in humans has already caused catastrophe around the globe (Shah et al. 2020). Meanwhile, no potential drugs or vaccines have been explored yet against this disease and additionally SARS-CoV-2 is extremely contagious as more than 2 healthy persons are being infected with a single pre-symptomatic and/or asymptomatic patient (Liu et al. 2020). To develop an impressive prevention and treatment plan toward COVID-19, a large number of laboratory based experiments are ongoing all over the world. The development of anti-COVID-19 treatment will take a few months to several years, which may worsen the present global pandemic situation. Therefore, in recent time, scientists are concentrating on the reuse of several existing drugs for treating COVID-19 pandemic. Numerous researches on drug repurposing approach have shed light on the use of a few well-known comprehensive antiviral drugs such as nucleoside analogue, HIV (human immunodeficiency virus) and HCV (hepatitis C virus) protease inhibitor. A small number of antiviral agents, i.e., lopinavir, ritonavir, oseltamivir, favinapir and remdesivir, have been tested clinically and recently have been used as a treatment regimen against COVID-19 (Devaux et al. 2020; Shah et al. 2020). A different protease inhibitor, namely camostat mesylate, has recently been reported as an anti-COVID-19 agent in humans and exerts its effect by inhibiting transmembrane protease serine 2 (TMPRSS2)-dependent viral entry into the host cell (Hoffmann et al. 2020). In the interim, two antimalarial agents, namely chloroquine and hydroxychloroquine, are already being administered to patients with emergency condition, and thought to play inhibitory function by binding with ACE2 receptor and acidifying the host cell membrane that ultimately prevents viral entry. Unfortunately, these antimalarials have some serious adverse impacts on patients with hypertension, diabetes, acute renal failure and cardiovascular disorder, and thus, were rejected by the Food and Drug Administration (FDA) as anti-COVID-19 therapeutics (Enmozhi et al. 2020).

Considering the lack of efficient therapeutics along with the constant increase in infection numbers and death cases, computer-assisted drug designing would be an eminent approach toward COVID-19 treatment. This structure-based rational drug design and development strategy will curtail the expense and time required for discovering novel drug candidates. According to the above-mentioned in silico technique, identification of a chemical compound (plant-derived and synthetic) as a potential cure depends on molecular docking and dynamics simulation of different chemicals from a known chemical library against target proteins (Meng et al. 2011; Gurung et al. 2020). The natural plant products are diversified chemical compounds collectively known as phytochemicals and can be classified into six major groups, namely carbohydrates, lipids, phenolic acids, alkaloids, terpenoids and other nitrogen containing metabolites. Furthermore, phenolic acids are then categorized into different subgroups, and among them flavonoids, tannins, stilbenes, lignans and quinones are the most remarkable (Gurung et al. 2020). These plant-derived phytochemicals are hardly toxic and much more safe than synthetic chemical compounds. They are considered as the rich source of efficient antiviral compounds, and about 44% of the total antiviral medications produced in between 1981 and 2006 were mostly derived from phytochemicals (Molyneux et al. 2007; Newman and Cragg 2007). Few examples of antiviral phytochemicals include tannins from *Phyllanthus amarus* found to inhibit HIV replication (Notka et al. 2004); a diterpenoid, namely andrographolide from *Andrographis paniculata*, shows inhibitory effect on the replication process of chikungunya, HSV-1 and dengue virus (Enmozhi et al. 2020); a flavonoid, i.e., fisetin (flavonol) from *Acacia nilotica*, have in vitro anti-HCV activity; a flavonoid, i.e., hesperetin (flavanone) from *Citrus aurantium*, displays antiviral activity against chikungunya, yellow fever virus and HSV-1 (Rehman et al. 2011).

Until now, no precise scheme has been established for the treatment/management of COVID-19. Repurposing of previously known novel antiviral phytochemicals would be a great strategy to tackle the deadly SARS-CoV-2. Therefore, in this experiment, a set of 43 flavonoids having well-known antiviral activity were chosen and assessed for their anti-COVID-19 potential through virtual ligand screening (VLS) technique. To achieve the goal, this study focused on targeting ACE2 of human host and Mpro/3CLpro and PLpro of SARS-CoV-2, hence impeding viral entry and maturation inside the host. The binding affinity of the selected flavonoids to the above-mentioned molecular targets was determined by using molecular docking and dynamics simulation approach.

## Methods

**Fig. 1:**
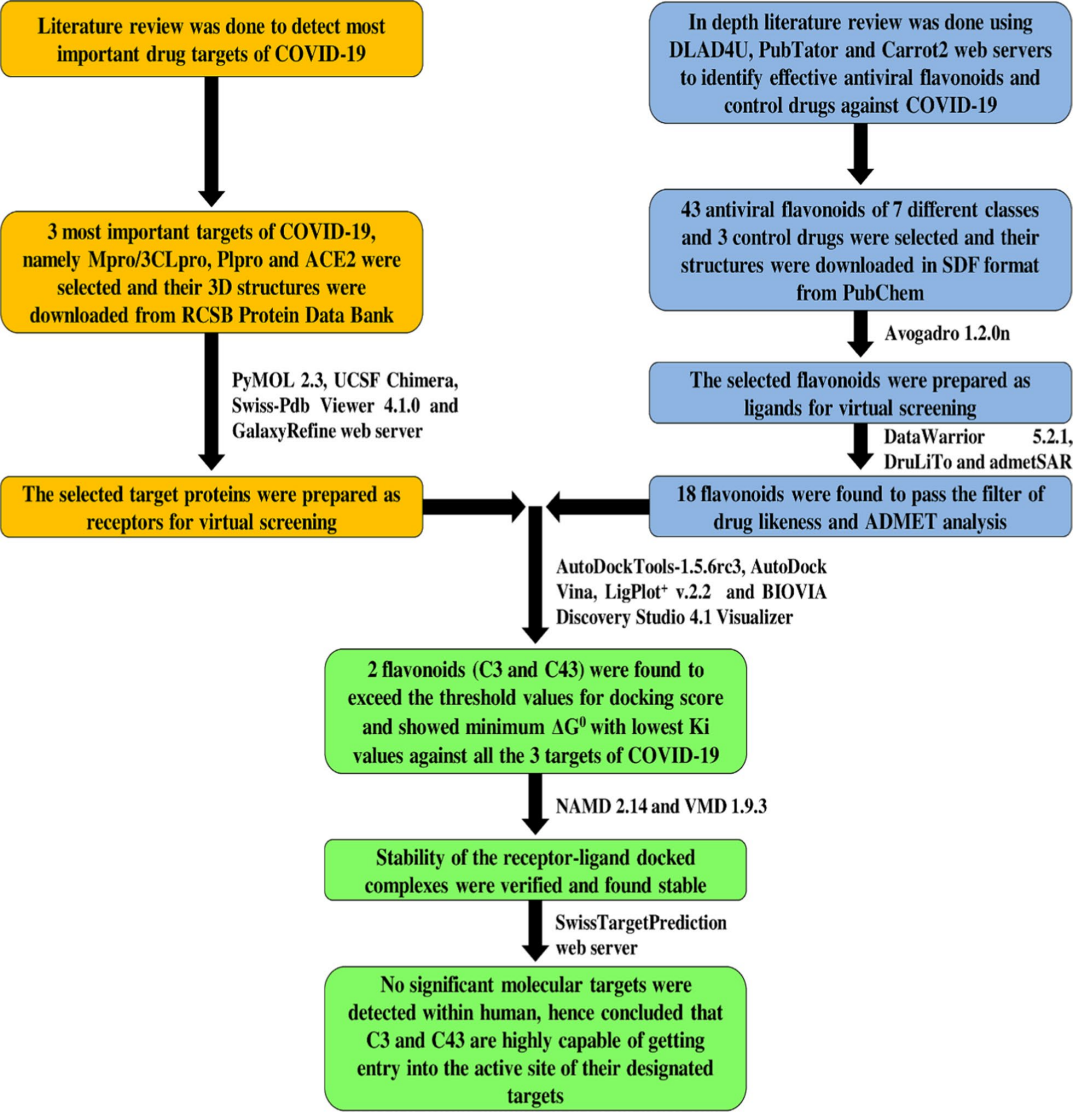
The complete workflow for the identification of luteolin and abyssinone II as most potent inhibitors against Mpro/3CLro, PLpro and ACE2 of COVID-19

Recent advancement in computational biology made virtual screening of natural bioactive compounds, i.e., phytochemicals, a gold standard technique in current drug discovery pipelines (Kitchen et al. 2004). In this research, a collection of flavonoids (phytochemicals) was chosen as potential inhibitors to execute computer-assisted site specific docking against Mpro/3CLpro, PLpro and ACE2 of COVID-19. The computational investigations were performed through a HP Pavilion 14-bf081tx laptop (Intel® core™ i7-7500 (7^th^ gen) CPU/8 GB of DDR4 RAM/64-bit OS of Windows 10). The complete methodology of this investigation is given in Fig. 1.

### Creation of flavonoids library

For repurposing previously known potent antiviral flavonoids against SARS-CoV-2, a set of 43 flavonoids consisting of different classes including flavones (*N* = 10), flavonols (*N* = 11), chalcones (*N* = 2), flavans (*N* = 4), isoflavones (*N* = 7), anthocyanidins (*N* = 5) and flavanones (*N* = 4) from different medicinal plants (Table 1) were fetched by an intensive literature search using DLAD4U, PubTator and Carrot2 web servers (Joshi et al. 2020). These bioactive compounds were selected on the basis of their earlier evidence for acting as antiviral agents against various human pathogenic viruses such as herpes simplex virus, dengue virus, human immunodeficiency virus, influenza virus, entero virus, ebola virus, rotavirus, rhinovirus, polio virus, zika virus, corona virus, hepatitis B and C virus. In addition, three recently applied antiviral drugs against COVID-19, namely hydroxychloroquine, remdesivir and camostat mesylate, were used as control (Shah et al. 2020; Uno 2020). The detailed information about selected flavonoids (class, ID, plant source, antiviral activity, mechanism of action and 2D structure) and control drugs is summarized in Additional file 1: Fig. S1 and Table S1.

**Table 1:** List of selected antiviral flavonoids (phytochemicals) and drugs used as control for docking against Mpro/3CLpro, PLpro and ACE2 of COVID-19

Flavonoid class	Name	Flavonoids	Flavonoid class	Name	Flavonoids
Flavones	C1	Apigenin	Flavans	C24	(+)-Catechin
	C2	Baicalein		C25	(−)-Epigallocatechin gallate
	C3	Luteolin		C26	(−)-Epicatechin gallate
	C4	Orientin		C27	(−)-Epigallocatechin
	C5	Vitexin	Isoflavones	C28	Genistein
	C6	Chrysosplenol C		C29	Glycitein
	C7	Wogonoside		C30	Daidzein
	C8	Chrysin		C31	Puerarin
	C9	Tangeretin		C32	Ononin
	C10	Wogonin		C33	Biochanin A
Flavonols	C11	Quercetin		C34	Formononetin
	C12	Kaempferol	Anthocyanidins	C35	Cyanidin
	C13	Rutin		C36	Peonidin
	C14	Fisetin		C37	Malvidin
	C15	Theaflavin		C38	Apigenidin
	C16	Luteoforol		C39	Delphinidin
	C17	Myricetin	Flavanones	C40	Eriodictyol
	C18	3-Methylkaempferol		C41	Hesperetin
	C19	Isorhamnetin		C42	Naringenin
	C20	Galangin		C43	Abyssinone II
	C21	Morin	Control	Con-1	Hydroxychloroquine
Chalcones	C22	Sappanchalcone		Con-2	Remdesivir
	C23	3-Deoxysappanchalcone		Con-3	Camostat mesylate

### Ligand preparation

The 3D chemical structures/conformers of preselected 43 flavonoids and 3 control drugs were downloaded from PubChem database in SDF (Spatial Data File) format (Shah et al. 2020). Polar hydrogen atoms were added into each of the chemical compound at pH 7.4 utilizing build function of Avogadro 1.2.0n software program. After that, geometry optimization followed by energy minimization was executed at MMFF94 force field along with conjugate gradients algorithm by utilizing the same program (Hanwell et al. 2012). These optimized ligand structures were saved into PDB (Protein Data Bank) file format for further analysis.

### Selection and preparation of receptor protein structures

The three-dimensional (3D) X-ray crystallographic structures of SARS-CoV-2 Mpro/3CLpro co-crystalized with inhibitor N3 (PDB id- 6LU7; single chain with a total of 306 amino acids), SARS-CoV-2 PLpro co-crystalized with peptide inhibitor VIR251 (PDB id- 6WX4; single chain with a total of 326 amino acids) and human ACE2 related carboxypeptidase co-crystalized with inhibitor XX5 (PDB id- 1R4L; single chain with a total of 615 amino acids) were retrieved from RCSB Protein Data Bank at a resolution of 2.16 Å, 1.66 Å and 3.0 Å, respectively (Additional file 1: Fig. S2) (Joshi et al. 2020; Rut et al. 2020). All of the previously bound ligands/inhibitors, ions and water molecules were selected and removed from those receptors by employing PyMOL 2.3 (Samofalova et al. 2017). After that, hydrogen atoms and charge were added to the proteins by applying the Dock Prep tool of UCSF Chimera 1.14 (Pettersen et al. 2004) and energy minimization was carried out using GROMOS96 program implemented in the Swiss-Pdb Viewer 4.1.0 (Johansson et al. 2012). The added hydrogens were optimized via an H-bonding network which had to determine the histidine protonation state. Finally, the energy minimized receptor structures were refined with GalaxyRefine web server (Enayatkhani et al. 2020) and used as receptor (in PDB format) for docking simulation purpose.

### Drug likeness/pharmacophore and ADMET profile analysis

Pharmacological significance of a particular ligand/chemical compound can be evaluated by analyzing different parameters like drug likeness/pharmacophore and ADMET properties. These characteristic features are determined by considering certain physically significant descriptors and pharmaceutically relevant properties of that particular compound. For predicting a proposed molecule as a potential drug candidate, pharmacokinetics and pharmacodynamics analysis are must and can be done with ADMET reasoning. To minimize undesired effects, ADMET analysis has a remarkable impact in pharma industries, and these days it is also extensively applied in computer aided drug designing (Elmezayen et al. 2020; Gurung et al. 2020; Joshi et al. 2020). In this experiment, all the phytochemicals (43 flavonoids) along with 3 drugs (used for control purpose) were scanned for drug likeness/pharmacophore features obeying Lipinski’s rule of five based on physicochemical properties (molecular weight, lipophilicity, water solubility, H-bond donor, H-bond acceptor, topological polar surface area and drug likeness). Thus, physicochemical properties of all the chemical compounds were discovered using SDF file as input by employing DataWarrior 5.2.1 and DruLiTo open source program (Gurung et al. 2020; Joshi et al. 2020). Thereafter, ADMET properties (blood brain barrier permeability, human intestinal absorption, Caco-2 permeability, AMES toxicity, carcinogenicity, mutagenicity, tumorigenicity, irritancy and reproductive effect) of all these ligands were tested by exploiting an online web server, namely admetSAR and DataWarrior 5.2.1 program (Elmezayen et al. 2020; Gurung et al. 2020). For admetSAR analysis, Simplified Molecular Input Line Entry System (SMILES) file format was used as input and entered into the search bar.

### Grid box generation, docking method validation and virtual screening

Structure-based virtual screening is an emerging and widely used efficient approach toward modern drug discovery that hunts for an effective chemical compound, i.e., ligand as candidate drug from a large library of small molecules and detects the complex interplay between essential amino acids and ligand (high binding affinity and low energy conformation) within the binding pocket of a drug/protein target (mainly enzyme and receptor) (Elmezayen et al. 2020; Shah et al. 2020). In this research, virtual screening in the form of focused molecular docking was performed using AutoDock Vina to explore the robust inhibitory effect of culled flavonoids (those which passed all the parameters of drug likeness and ADMET analysis) on Mpro/3CLpro, PLpro and ACE2 against COVID-19. A previous study reported that focused molecular docking approach is much more precise than blind molecular docking approach (Ghersi and Sanchez 2009). Relying on Broyden–Fletcher–Goldfarb–Shanno algorithm along with empirical and knowledge-based scoring functions, AutoDock Vina provides accurate and high performance docking score with possible orientations and conformations for a particular ligand at a binding site (Elmezayen et al. 2020). To authenticate molecular docking protocol and algorithm, a re-docking experiment was carried out in this study to imitate the native binding poses with the co-crystal reference compound (inhibitor) N3, VIR251 and XX5 into the binding pocket of 6LU7 (Mpro/3CLpro), 6WX4 (PLpro) and 1R4L (ACE2), respectively. Later on, root mean square deviation (RMSD) values for docked ligands with respect to reference ligands at the crystal structures were detected using BIOVIA Discovery Studio 4.1 Visualizer (Elmezayen et al. 2020). The 3D grid boxes were constructed with suitable dimensions using AutoDockTools-1.5.6rc3, which analyzes the active sites within the crystal structures bound by co-crystal ligands (Table 2) (Joshi et al. 2020). The outcome of re-docking experiment demonstrated that the docked and co-crystalized reference molecules were partially superimposed to each other (RMSD value was < 3.0 Å between docked and co-crystalized reference ligands); therefore, the docking method was considered sufficient enough for virtual screening. Thereon, rigid molecular docking simulation was performed with AutoDock Vina at a search space volume 27,000 Å^3^ and exhaustiveness heuristics 8 (E = 8) between receptor/target proteins and selected compounds (flavonoids as well as reference molecules and control drugs), in which the ligands were kept flexible while the receptors were kept rigid at all the time. Prior to virtual screening, torsional readjustment was done with the ligands that made rotatable torsion during molecular docking (Rasool et al. 2018). In order to anticipate the binding affinity and ligand efficiency of a distinct chemical compound as inhibitor of COVID-19, five separate docking runs were executed against each of the three SARS-CoV-2 targets. Thus, a total of 360 (24 × 3 × 5) independent docking runs were performed in this study. Finally, the interactions between flavonoids and target proteins with most appropriate binding conformations were evaluated by assessing minimum binding free energies/Gibbs free energy (Δ*G*^0^ in kcal/mol) and lowest inhibition/dissociation constant (Ki in nm) which could be determined by Eqs. 1 and 2, respectively. It is already established that stable protein ligand complex displays more negative Δ*G*^0^, while significant inhibitory potential is exhibited by minimum Ki (Gurung et al. 2020).

1 $$\Delta G^{0} = \, a \times {\text{vdW}} + b \times {\text{Coul}} + {\text{Hbond}} + {\text{Metal}} + {\text{Lipo}} + {\text{BuryP}} + {\text{RotB}} + {\text{Site}}$$

where *a* = coefficient constant for vdW, *b* = coefficient constant for Coul, vdW = van der Waals energy, Coul = Coulomb energy, Hbond = hydrogen bonding with receptor, Metal = binding with metal, Lipo = constant term for lipophilic, BuryP = buried polar group penalty, RotB = rotatable bond penalty and Site = active site polar interaction (Shah et al. 2020).

2 $${\text{Ki}} = {\text{exponential }}\left( {\Delta G^{0} /RT} \right)$$

where *R* = universal gas constant (1.987 cal K^−1^ mol^−1^) and *T* = temperature (298.15 K) [Gurung et al. 2020].

**Table 2:** Grid box coordinates used in AutoDock Vina for molecular docking studies against Mpro/3CLpro, PLpro and ACE2 of COVID-19

Target protein (PDB id)	Grid box dimension		
	Number of grid points (*x* × *y* × *z* dimension)	Center grid box (*xyz* coordinates)	Grid point spacing (Å)
Mpro/3CLpro (6LU7)	55 × 55 × 55	− 10.78, 11.69, 68.89	0.375
PLpro (6WX4)	40 × 40 × 40	9.67, − 27.06, − 37.17	0.375
ACE2 (1R4L)	35 × 35 × 35	40.98, 6.53, 28.33	0.375

In the current research, threshold values for binding free energy and inhibition constant were set on the basis of average Δ*G*^0^ and Ki scores of docked reference compounds and drugs (used as control) against Mpro/3CLpro, PLpro and ACE2. The threshold values were applied to minimize the search area and hit compounds having lower/minimum scores than threshold in terms of both Δ*G*^0^ and Ki values.

### Visualization and interaction pattern analysis

The 2D and 3D visualization of non-bonded interactions within ligand–receptor docked complex were done by LigPlot^+^ v.2.2 and BIOVIA Discovery Studio 4.1 Visualizer (Rahman et al. 2016; Joshi et al. 2020; Umar et al. 2020). The indicated tools are capable of identifying different interaction patterns (H-bonds, hydrophobic and electrostatic interactions with their respective bond lengths) between an amino acid residue of a receptor and a ligand molecule.

### Molecular dynamics (MD) simulation

Molecular dynamics is an advanced computer-assisted simulation method that is generally utilized for analyzing the stability of a receptor-ligand docked complex at microscopic/atomic level by expressing the behavioral property, interaction pattern, physical basis of function, solvation property, structural property, fluctuation and conformational changes of the docked complex (Snøve and Holen 2004; Song et al. 2004). In this experiment, MD simulation was used as a validation technique for the docking results of top ranked ligand molecules and control drugs having average Δ*G*^0^ and Ki scores above the threshold with the 3 COVID-19 targets (Mpro/3CLpro, PLpro and ACE2). The stability of the different docked complexes was examined by trajectory analysis with the help of NAMD 2.14 (NAMD_2.14bNAMD_2.14b2_Win64-multicore-CUDA version) graphical interface module with CHARMM36 as a force filed integrated with VMD 1.9.3 program (Phillips et al. 2005; MacKerell et al. 1998). Visual molecular dynamics (VMD) was employed to create water box, neutralize the system and generate PSF (Protein Structure File) files of the docked complexes (Humphrey et al. 1996), whereas CHARMM-GUI web based graphical interface was adopted to construct ligand topology and parameter files (Jo et al. 2008). The simulation was run for 5 ns (nanosecond)/5000 ps (picosecond) keeping a constant temperature of 310 K by using a Langevin thermostat. The system was minimized for 500 steps. Periodic boundary conditions and time step of 2 fs (femtosecond) was used for the simulation. At the end, stability of the docked complexes was determined by evaluating the changes in RMSD and root mean square fluctuation (RMSF) values of the system (Nosrati et al. 2019).

### Molecular target anticipation for selected flavonoids

Current research on drug designing and/or development remarkably depends on different studies that predict molecular targets for a particular chemical compound within a biological entity. These experiments are very critical to identify probable cross-reactivity or adverse side effects within an organism, i.e., *H. sapiens* induced by the activity of small bioactive compounds (Enmozhi et al. 2020). For this purpose, SwissTargetPredcition web server was adopted to determine the molecular targets within humans for flavonoids which had already passed the threshold barrier for molecular docking results and satisfied MD simulation analysis (Daina et al. 2019). Inside the search bar, structure of the two flavonoids in the form of canonical SMILES format was used as input and analyzed.

## Results and discussion

### Screening of drug likeness/pharmacophore and ADMET features

In the field of drug design and development, there is always a high failure rate for a proposed chemical compound to be used as an effective drug candidate in preclinical and clinical trials due to poor pharmacokinetic studies. Hence, drug likeness/pharmacophore and ADMET profile analysis of that specific compound may increase the chances of passing through the preclinical/clinical trials (Gurung et al. 2020). Within this experiment, characteristics evaluation of drug likeness/pharmacophore was done utilizing two open source software programs, namely DataWarrior 5.2.1 and DruLiTo, whereas ADMET was done adopting a freely accessible web server, namely admetSAR. After inspecting the drug likeness/pharmacophore and ADMET properties of 43 preselected flavonoid phytochemicals, a total of 18 molecules (three active flavones, namely C2/baicalein, C3/luteolin and C8/chrysin; two active flavonols, namely C16/luteoforol and C18/3-methylkaempferol; two active chalcones, namely C22/sappanchalcone and C23/3-deoxysappanchalcone; one active flavan, namely C24/( +)-catechin; two active isoflavones, namely C33/biochanin A and C34/formononetin; four active anthocyanidins, namely C35/cyanidin, C36/peonidin, C37/malvidin and C38/apigenidin and four active flavanones, namely C40/eriodictyol, C41/hesperetin, C42/naringenin and C43/abyssinone II) were found to be orally bioactive (Table 3). The above-mentioned flavonoids were also found to be drug-like compounds obeying Lipinski’s rule of five with no violation (molecular weight (MW) ≤ 500 Da, Log *P* (a measure of lipophilicity) ≤ 5, number of hydrogen bond donors (HBD) ≤ 5 and number of hydrogen bond acceptors (HBA) ≤ 10) and fall within the satisfactory range for solubility (Log *S*, between − 6.5 and 0.5), topological polar surface area (TPSA) ≤ 140 Å^2^, molar refractivity (a measure of the total polarizability of a mole of a given substance) ≤ 130 and drug likeness score (between − 7 and + 7) (Guan et al. 2018). In terms of toxicity, none of those compounds showed significant toxicity issues such as AMES toxic, carcinogenic, mutagenic, tumorigenic, irritant and adverse effects on reproductive health. With respect to absorption through biological membrane, five (C8, C23, C34, C38 and C42) out of 18 flavonoids showed complete permeability between blood brain barrier, human intestinal epithelium and caco-2 (colorectal carcinoma) cell. These phytochemicals having drug like/pharmacophore activities were further considered for molecular docking studies. Subsequent drug likeness and ADMET properties analysis of the 3 control medications showed interesting results. Control drug-2/remdesivir was found to violate Lipinski’s rule of five with substantial toxicity effects, i.e., tumorigenic, irritant and injurious for reproductive system, whereas control drug-1/hydroxychloroquine and 3/camostat mesylate were found to be AMES positive, carcinogenic and mutagenic.

**Table 3:** Drug likeness/pharmacophore and ADMET profile of screened antiviral flavonoids (phytochemicals) along with drugs used as control for docking against Mpro/3CLpro, PLpro and ACE2 of COVID-19

Name	Drug likeness/pharmacophore features									ADMET properties								
	MW^a^ (Da)	Log *P*^b^	Log *S*^c^	HBA^d^	HBD^e^	TPSA^f^ (Å^2^)	Molar refractivity	Drug likeness	Violation of Lipinski’s rule of five	BBB^g^ permeability	HIA^h^	Caco-2 permeability	AMES toxicity	Carcinogenic	Mutagenic	Tumorigenic	Irritant	Reproductive effect
C1	270.05	1.14	− 2.86	5	3	86.99	73.99	0.28	No	Yes	Yes	Yes	No	No	Yes	No	No	None
C2*	270.05	2.63	− 2.86	5	3	86.99	73.99	0.28	No	No	Yes	No	No	No	No	No	No	None
C3*	286.05	1.49	− 2.86	6	4	107.22	76.01	0.28	No	No	Yes	No	No	No	No	No	No	None
C4	448.1	− 0.36	− 1.97	11	8	197.37	108.63	− 2.0	Yes	No	Yes	No	Yes	No	Yes	No	No	None
C5	432.11	− 0.71	− 2.27	10	7	177.14	106.61	− 2.0	Yes	No	Yes	No	Yes	No	Yes	No	No	None
C6	360.08	2.29	− 2.96	8	3	114.68	93.47	− 0.16	No	No	Yes	Yes	No	No	Yes	No	No	None
C7	460.1	0.94	− 3.04	11	5	172.21	111.19	0.5	Yes	No	Yes	No	No	No	Yes	Yes	No	None
C8*	254.06	1.85	− 3.15	4	2	66.76	71.97	0.28	No	Yes	Yes	Yes	No	No	No	No	No	None
C9	372.12	2.58	− 3.83	7	0	72.45	100.38	0.36	No	Yes	Yes	Yes	No	No	Yes	Yes	No	None
C10	284.07	2.09	− 3.17	5	2	75.99	78.46	0.4	No	No	Yes	Yes	No	No	Yes	No	No	None
C11	302.04	1.83	− 2.49	7	5	127.45	78.03	− 0.08	No	No	Yes	No	No	No	Yes	Yes	No	None
C12	286.05	1.49	− 2.79	6	4	107.22	76.01	− 0.08	No	Yes	Yes	No	No	No	Yes	No	No	None
C13	610.15	− 0.74	− 2.4	16	10	265.52	141.4	1.93	Yes	No	Yes	No	No	No	No	No	No	None
C14	286.05	1.92	− 2.79	6	4	107.22	76.01	− 0.08	No	Yes	Yes	No	No	No	Yes	No	No	None
C15	564.13	0.88	− 3.93	12	9	217.6	143.98	1.46	Yes	No	Yes	No	No	No	No	No	No	None
C16*	290.08	1.12	− 1.62	6	5	110.38	74.33	− 0.07	No	No	Yes	No	No	No	No	No	No	None
C17	318.04	2.18	− 2.19	8	6	147.68	80.06	− 0.08	Yes	No	Yes	No	No	No	Yes	No	No	None
C18*	300.06	1.38	− 2.91	6	3	96.22	80.48	− 0.11	No	No	Yes	Yes	No	No	No	No	No	None
C19	316.06	1.73	− 2.81	7	4	116.45	82.5	0.06	No	No	Yes	Yes	No	No	Yes	No	No	None
C20	270.05	2.2	− 3.08	5	3	86.99	73.99	− 0.08	No	Yes	Yes	No	No	No	Yes	No	No	None
C21	302.04	1.41	− 2.49	7	5	127.45	78.03	− 0.08	No	Yes	Yes	No	No	No	Yes	No	No	None
C22*	286.08	1.69	− 2.97	5	3	86.99	78.81	0.22	No	No	Yes	Yes	No	No	No	No	No	None
C23*	270.09	1.56	− 3.27	4	2	66.76	76.79	0.22	No	Yes	Yes	Yes	No	No	No	No	No	None
C24*	290.08	0.85	− 1.76	6	5	110.38	74.33	0.32	No	No	Yes	No	No	No	No	No	No	None
C25	458.08	2.98	− 2.16	11	8	197.37	112.06	− 0.33	Yes	No	Yes	No	No	No	No	No	No	None
C26	442.09	2.64	− 2.46	10	7	177.14	110.04	− 0.33	Yes	No	Yes	No	No	No	No	No	No	None
C27	306.07	1.2	− 1.47	7	6	130.61	76.36	0.32	Yes	No	Yes	No	No	No	No	No	No	None
C28	270.05	1.04	− 2.72	5	3	86.99	73.99	− 0.09	No	Yes	Yes	Yes	No	No	Yes	Yes	No	Yes
C29	284.07	1.36	− 3.04	5	2	75.99	78.46	0.04	No	No	Yes	Yes	No	No	No	No	No	Yes
C30	254.06	1.12	− 3.02	4	2	66.76	71.97	− 0.09	No	Yes	Yes	Yes	No	No	No	No	No	Yes
C31	416.11	− 0.72	− 2.44	9	6	156.91	104.59	− 2.4	Yes	No	Yes	No	Yes	No	No	No	No	Yes
C32	430.13	0.14	− 3.22	9	4	134.91	108.56	− 3.44	No	No	Yes	No	No	No	No	Yes	No	None
C33*	284.07	1.36	− 3.04	5	2	75.99	78.46	0.04	No	No	Yes	Yes	No	No	No	No	No	None
C34*	268.07	1.45	− 3.34	4	1	55.76	76.43	0.04	No	Yes	Yes	Yes	No	No	No	No	No	None
C35*	287.06	2.41	− 3.25	6	5	101.15	76.17	− 2.12	No	Yes	Yes	No	No	No	No	No	No	None
C36*	301.07	1.86	− 3.57	6	4	90.15	80.64	− 5.89	No	No	Yes	Yes	No	No	No	No	No	None
C37*	331.08	2.1	− 3.57	7	4	99.38	87.13	− 5.89	No	No	Yes	Yes	No	No	No	No	No	None
C38*	290.03	2.21	− 3.85	4	3	60.69	77.98	− 6.14	No	Yes	Yes	Yes	No	No	No	No	No	None
C39	338.02	2.83	− 2.96	7	6	121.38	84.05	− 6.14	Yes	Yes	Yes	No	No	No	No	No	No	None
C40*	288.06	1.14	− 2.34	6	4	107.22	73.59	− 0.22	No	No	Yes	No	No	No	No	No	No	None
C41*	302.08	1.03	− 2.66	6	3	96.22	78.06	− 0.09	No	No	Yes	Yes	No	No	No	No	No	None
C42*	272.07	0.79	− 2.64	5	3	86.99	71.57	− 0.22	No	Yes	Yes	Yes	No	No	No	No	No	None
C43*	324.14	2.49	− 3.86	4	2	66.76	93.27	− 0.34	No	No	Yes	Yes	No	No	No	No	No	None
Con-1	335.18	1.55	− 3.55	4	2	47.86	98.57	5.73	No	Yes	Yes	No	Yes	No	Yes	No	No	None
Con-2	602.33	0.34	− 4.99	14	4	211.13	150.43	− 21.38	Yes	No	Yes	No	No	No	No	Yes	Yes	Yes
Con-3	398.16	− 0.72	− 2.99	9	2	137.31	123.31	2.7	No	Yes	No	No	No	Yes	No	No	No	None

*Indicates the flavonoids which passed all the following parameters

^a^Molecular weight

^b^Partition coefficient between n-octanol and water

^c^Water solubility

^d^Hydrogen bond acceptor

^e^Hydrogen bond donor

^f^Topological polar surface area

^g^Blood Brain Barrier

^h^Human intestinal absorption

### Docking method verification and structure-based virtual screening

**Fig. 2:**
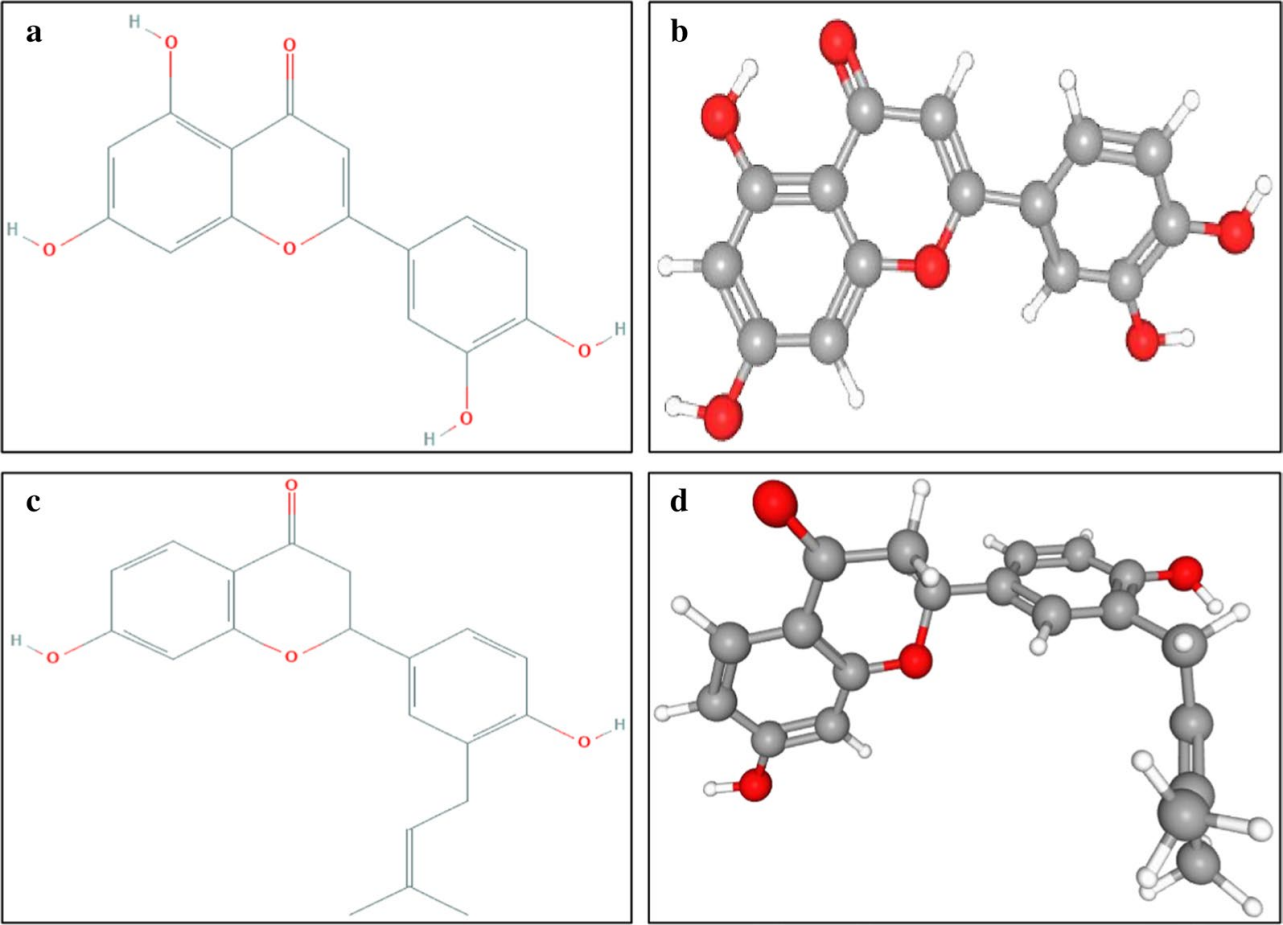
**a** 2D structure of C3/luteolin, **b** 3D structure of C3/luteolin, **c** 2D structure of C43/abyssinone II and **d** 3D structure of C43/abyssinone II

Before performing docking simulations for virtual screening, the docking procedure and algorithm were validated using a re-docking experiment between original co-crystal reference molecules (N3, VIR251 and XX5) and three different COVID-19 targets (Mpro/3CLpro, PLpro and ACE2). The re-docking report revealed that reference inhibitor N3, VIR251 and XX5 had an RMSD value of 2.873 Å, 2.328 Å and 2.761 Å, respectively, between the docked and native co-crystal conformation. Recent findings suggest that docking solution having RMSD value ≤ 2.0 Å, 2.0 Å—3.0 Å and ≥ 3.0 Å is considered as good, acceptable and bad solution, respectively (Ramírez and Caballero 2018). Hence, slight deviation in RMSD implies that the molecular docking protocol, parameters and algorithm used within this experiment were reliable enough to mimic the biological conformations of the molecules (Gurung et al. 2020). AutoDock Vina was considered as the main platform for virtual screening and assessing the ligand binding efficiency of native reference compounds N3, VIR251 and XX5 extracted from the crystal structure of 6LU7, 6WX4 and 1R4L, respectively. Simultaneously, the binding affinity of 3 control drugs (hydroxychloroquine, remdesivir and camostat mesylate) was also evaluated against three COVID-19 targets. The binding free energy, Δ*G*^0^ along with inhibition constant, Ki score (stated in bracket) of N3 with Mpro/3CLpro, VIR251 with PLpro and XX5 with ACE2 was found to be − 6.9 kcal/mol (8391.94 nM), − 5.5 kcal/mol (89,909.02 nM) and − 8.2 kcal/mol (927.87 nM), respectively (Table 4). Hydroxychloroquine had a Δ*G*^0^ (Ki) value of − 5.8 kcal/mol (54,088.09 nM), − 5.4 kcal/mol (106,505.11 nM) and − 7.7 kcal/mol (2164.34 nM) for Mpro/3CLpro, PLpro and ACE2, respectively. At the same time, remdesivir was found to have a Δ*G*^0^ (Ki) value of − 6.7 kcal/mol (11,775.97 nM), − 6.5 kcal/mol (16,524.61 nM) and − 10.0 kcal/mol (43.98 nM) against Mpro/3CLpro, PLpro and ACE2, respectively. The binding affinity and inhibition constant of camostat mesylate showed similar pattern like remdesivir bearing Δ*G*^0^ (Ki) value of − 6.7 kcal/mol (11,775.97 nM), − 5.9 kcal/mol (45,659.84 nM) and − 9.0 kcal/mol (239.3 nM) against Mpro/3CLpro, PLpro and ACE2, respectively. Hydroxychloroquine displayed lowest binding affinity against all the three targets compared to different flavonoids and other 2 controls, while remdesivir showed highest binding affinity compared to hydroxychloroquine and camostat mesylate. After acknowledging the binding scores of different reference molecules and control drugs, threshold for binding free energy was set − 8.0 kcal/mol, − 7.0 kcal/mol and − 10.0 kcal/mol for Mpro/3CLpro, PLpro and ACE2, respectively. Thus, the benchmark applied in virtual screening assured that molecular docking simulation was ended up with particular flavonoids/ligands having highest binding affinity in terms of minimum Δ*G*^0^ and lowest Ki value to their respective targets. After analyzing the binding affinity of top 18 molecules shown in Table 3, C3/Luteolin (PubChem CID: 5280445) (Fig. 2a, b) and C43/Abyssinone II (PubChem CID: 10064832) (Fig. 2c, d) were found to exceed the threshold values and showed minimum binding free energy and lowest inhibition constant toward all of the three COVID-19 targets. Luteolin is a flavone which can be isolated from medicinal plant *Ocimum basilicum*, *Spinacia oleracea* and *Capsicum annuum* and have in vitro antiviral activity against a wide range of viruses including HIV-1, EBV, EV71, SARS-CoV, influenza virus and JEV (Zakaryan et al. 2017). Alternately, abyssinone II is a flavanone found in *Citrus reticulate* and *Prunus cerasus* plant with in vitro inhibitory activity against influenza virus (Mohammadi Pour et al. 2019). Luteolin displays antiviral activity by inhibiting RNA replication and viral reactivation, while the mechanism of antiviral activity of abyssinone II is not detected yet (Additional file 1: Table S3). One of the two outranked flavonoids, i.e., abyssinone II, showed highest binding affinity with minimum Δ*G*^0^ and lowest Ki value (stated in bracket) of − 8.4 kcal/mol (661.23 nM), − 7.3 kcal/mol (4261.8 nM) and − 10.5 kcal/mol (18.86 nM) for Mpro/3CLpro, PLpro and ACE2, respectively, whereas luteolin had a Δ*G*^0^ (Ki) value of − 8.2 kcal/mol (927.87 nM), − 7.1 kcal/mol (5980.37 nM) and − 10.1 kcal/mol (37.13 nM) for Mpro/3CLpro, PLpro and ACE2, respectively (Table 4).

**Table 4:** Computed binding free/Gibbs free energy (Δ*G*^0^) and calculated inhibition/dissociation constant (Ki) scores of reference ligands, selected flavonoids and drugs (used as control) against Mpro/3CLpro, PLpro and ACE2 of COVID-19

Name	Flavonoids	Mpro/3CLpro (6LU7)		PLpro (6WX4)		ACE2 (1R4L)	
		Binding energy, Δ*G*^0^ (kcal/mol)	Inhibition constant, Ki (nM)	Binding energy, Δ*G*^0^ (kcal/mol)	Inhibition constant, Ki (nM)	Binding energy, Δ*G*^0^ (kcal/mol)	Inhibition constant, Ki (nM)
N3	–	− 6.9	8391.94	–	–	–	–
VIR251	–	–	–	− 5.5	89,909.02	–	–
XX5	–	–	–	–	–	− 8.2	927.87
C2	Baicalein	− 6.9	8391.94	− 6.2	27,468.36	− 8.9	283.48
C3	Luteolin	− 8.2	927.87	− 7.1	5980.37	− 10.1	37.13
C8	Chrysin	− 6.8	9940.99	− 6.3	23,188.12	− 8.6	471.22
C16	Luteoforol	− 7.2	5048.48	− 6.1	32,538.68	− 8.7	397.79
C18	3-Methylkaempferol	− 6.9	8391.94	− 6.0	38,544.92	− 8.4	66,123
C22	Sappanchalcone	− 6.6	13,949.67	− 5.9	45,659.84	− 8.6	471.22
C23	3-Deoxysappanchalcone	− 6.2	27,468.36	− 5.7	64,072.08	− 8.2	927.87
C24	(+)-Catechin	− 7.0	7084.27	− 6.4	19,574.84	− 8.5	558.19
C33	Biochanin A	− 6.8	9940.99	− 6.8	9940.99	− 8.9	283.48
C34	Formononetin	− 7.0	7084.27	− 6.4	19,574.84	− 8.9	283.48
C35	Cyanidin	− 7.1	5980.37	− 6.6	13,949.67	− 8.9	283.48
C36	Peonidin	− 6.9	8391.94	− 6.6	13,949.67	− 9.0	239.3
C37	Malvidin	− 6.8	9940.99	− 5.8	54,088.09	− 8.8	335.8
C38	Apigenidin	− 6.7	11,775.97	− 6.4	19,574.84	− 8.7	397.79
C40	Eriodictyol	− 7.5	3037.1	− 6.1	32,538.68	− 8.8	335.8
C41	Hesperetin	− 7.3	4261.8	− 6.1	32,538.68	− 8.8	335.8
C42	Naringenin	− 7.1	5980.37	− 6.2	27,468.36	− 8.6	471.22
C43	Abyssinone II	− 8.4	661.23	− 7.3	4261.8	− 10.5	18.86
Con-1	Hydroxychloroquine	− 5.8	54,088.09	− 5.4	106,505.11	− 7.7	2164.34
Con-2	Remdesivir	− 6.7	11,775.97	− 6.5	16,524.61	− 10.0	43.98
Con-3	Camostat mesylate	− 6.7	11,775.97	− 5.9	45,659.84	− 9.0	239.3

### Comparing the binding affinity between flavonoids and control drugs

The binding free energies (Δ*G*^0^) and inhibition constants (Ki) of 18 different flavonoids against three different targets of COVID-19 (Mpro/3CLpro, PLpro and ACE2) were compared with a batch of three previously used drugs against SARS-CoV-2, namely hydroxychloroquine, remdesivir and camostat mesylate. Hydroxychloroquine was first used as an antimalarial medication, and these days, it has also been applied to treat the symptoms of rheumatoid arthritis. Intensive research has already been conducted to examine the inhibitory effects of this drug against COVID-19. However, recent clinical trials in mid-2020 suggest that it is inefficient against SARS-CoV-2 and may cause harmful/unwanted secondary effects. The mechanism of action of hydroxychloroquine is not fully known to us. It is believed that this drug increases the lysosomal pH in antigen presenting cells and blocks toll-like receptors activation in plasmacytoid dendritic cells and thus reduces the activation of inflammatory process (Meyerowitz et al. 2020). The drug remdesivir (an analogue of adenosine triphosphate) was first invented to use as an anti-HCV agent and was then checked against ebola and marburg virus. However, it was found to be ineffective toward those viral infections (Mehta et al. 2020). Presently, this drug is being tested as an anti-COVID-19 agent by different research groups around the globe and has recently been approved by the USA, Singapore, India, Japan, the UK, the European Union and Australia for emergency use to patients with severe symptoms. The antiviral activity of remdesivir is exhibited by the inactivation of RNA-dependent RNA polymerase and exoribonuclease, which ultimately results in decreased viral RNA production (Mehta et al. 2020). In 1980s, camostat mesylate was first developed in Japan as protease inhibitor to treat acute symptoms of chronic pancreatitis and postoperative reflux esophagitis (Uno 2020). In present day, this drug is used as an anti-COVID-19 medication due to its suppressing power toward TMPRSS2 (a serine protease), which primes the spike protein of SARS-CoV-2 to ACE2 and facilitates viral entry into the host (Uno 2020). After thorough scrutinization, it was found that the Δ*G*^0^ and Ki values of different flavonoids showed inconsistency toward respective targets compared to the same values of the 3 control drugs (Table 4). For Mpro/3CLpro, a total of 15 flavonoids C2/baicalein, C3/luteolin, C8/chrysin, C16/luteoforol, C18/3-methylkaempferol, C24/(+)-catechin, C33/biochanin A, C34/formononetin, C35/cyanidin, C36/peonidin, C37/malvidin, C40/eriodictyol, C41/hesperetin, C42/naringenin and C43/abyssinone II were found to have higher binding affinity in terms of lower Δ*G*^0^ and Ki scores compared to the controls. On the contrary, five flavonoids C3/luteolin, C33/biochanin A, C35/cyanidin, C36/peonidin and C43/abyssinone II showed lower Δ*G*^0^ and Ki values against PLpro than the control drugs. On the other hand, 2 flavonoids C3/luteolin and C43/abyssinone II displayed higher binding affinity with ACE2 compared to the controls.

### Evaluating the interaction pattern between ligands and receptors

**Fig. 3:**
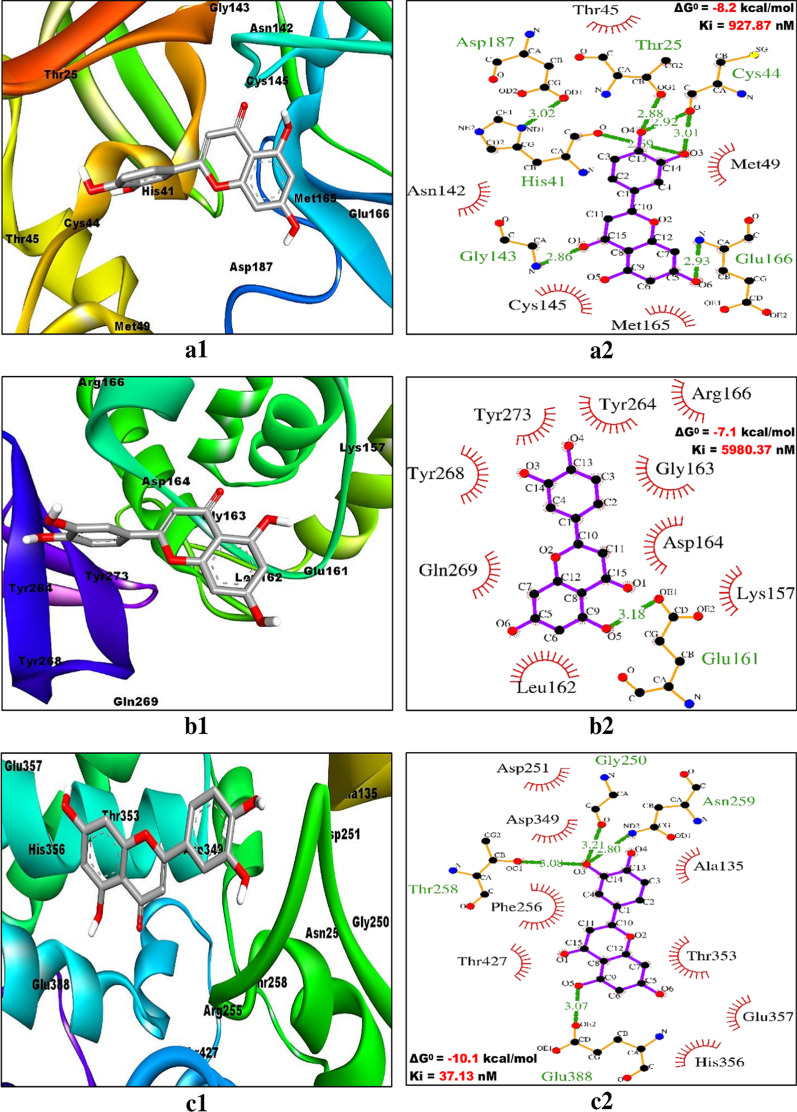
Binding poses (within the active site) and molecular interaction between C3/luteolin and different targets of COVID-19. **a1**, **a2** C3/luteolin and Mpro/3CLpro, **b1**, **b2** C3/luteolin and PLpro and **c1**, **c2** C3/luteolin and ACE2. Inside the active site, targets are illustrated as solid ribbon and the bound ligands as stick. The molecular interactions are represented by the green dashed lines for hydrogen bonds and semi-arcs with red eyelashes for hydrophobic interactions

**Fig. 4:**
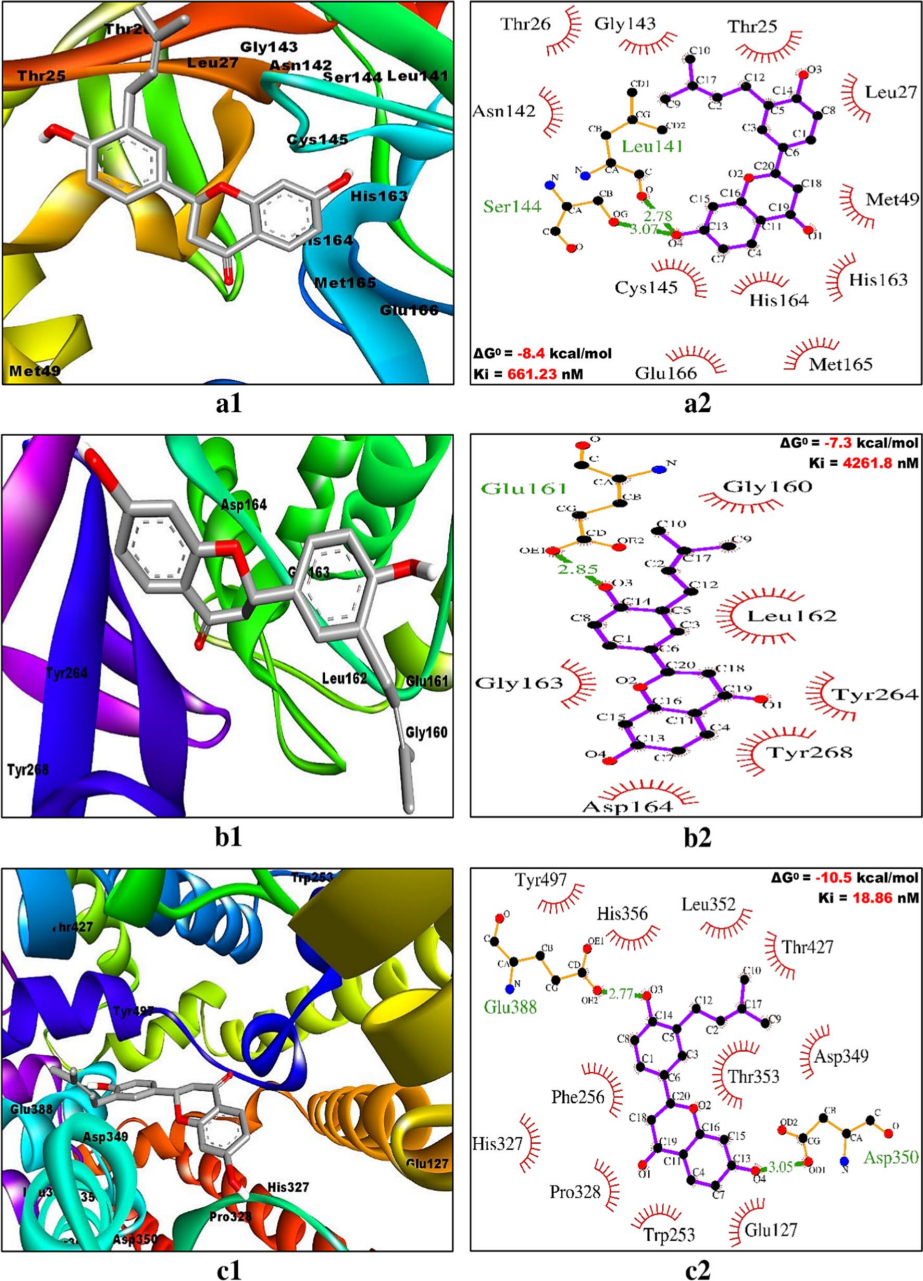
Binding poses (within the active site) and molecular interaction between C43/abyssinone II and different targets of COVID-19. **a1**, **a2** C43/abyssinone II and Mpro/3CLpro, **b1**, **b2** C43/abyssinone II and PLpro and **c1**, **c2** C43/abyssinone II and ACE2. Inside the active site, targets are illustrated as solid ribbon and the bound ligands as stick. The molecular interactions are represented by the green dashed lines for hydrogen bonds and semi-arcs with red eyelashes for hydrophobic interactions

**Fig. 5:**
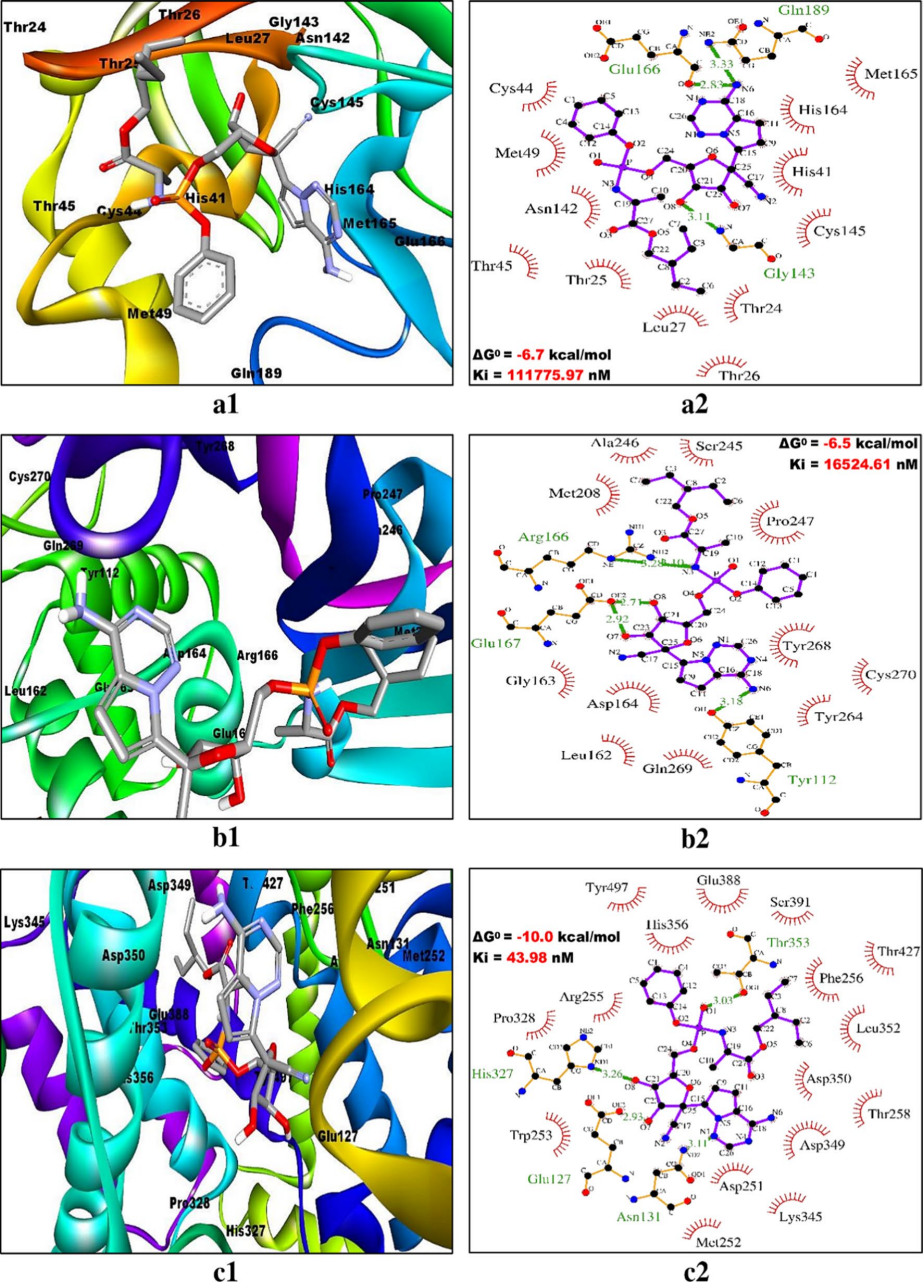
Binding poses (within the active site) and molecular interaction between Con-2/remdesivir and different targets of COVID-19. **a1**, **a2** Con-2/remdesivir and Mpro/3CLpro, **b1**, **b2** Con-2/remdesivir and PLpro and **c1**, **c2** Con-2/remdesivir and ACE2. Inside the active site, targets are illustrated as solid ribbon and the bound ligands as stick. The molecular interactions are represented by the green dashed lines for hydrogen bonds and semi-arcs with red eyelashes for hydrophobic interactions

The two-dimensional interactions of the best ranked flavonoids (C3/luteolin and C43/abyssinone II) and the top control drug (Con-2/remdesivir) at the active binding pockets of Mpro/3CLpro, PLpro and ACE2 were assessed by using LigPlot^+^ v.2.2 software program. All the restraining conformations of different ligands within the active sites of three receptors were found to be established with both the conventional hydrogen bonds (H-bonds) and hydrophobic interactions. The details of interacting atoms and amino acid residues associated with bond formation (both H-bonds and hydrophobic interactions) along with their corresponding bond length (only H-bonds) are given in Table 5. The binding of C3/luteolin to the active pocket of Mpro/3CLpro, PLpro and ACE2 was mediated by 6 H-bonds along with 20 hydrophobic interactions, 1 H-bond as well as 25 hydrophobic interactions and 4 H-bonds plus 31 hydrophobic interactions, respectively (Fig. 3). Meanwhile, flavonoid C43/abyssinone II did bind to Mpro/3CLpro, PLpro and ACE2 through 2 H-bonds together with 23 hydrophobic interactions, 1 H-bond in addition to 29 hydrophobic interactions and 2 H-bonds as well as 28 hydrophobic interactions, respectively (Fig. 4). Simultaneously, the interaction of control drug Con-2/remdesivir was found to be strengthened with Mpro/3CLpro, PLpro and ACE2 by the formation of 3 H-bonds plus 17 hydrophobic interactions, 5 H-bonds along with 19 hydrophobic interactions and 4 H-bonds together with 25 hydrophobic interactions, respectively (Fig. 5). Thus, interpreting the results for docking simulation and interaction pattern analysis, C3/luteolin and C43/abyssinone II were disclosed as potent inhibitors with enhanced binding affinity compared to remdesivir against Mpro/3CLpro, PLpro and ACE2 of COVID-19.

**Table 5:** Interaction pattern analysis of two top ranked flavonoids (C3/luteolin and C43/abyssinone II) along with top control drug (Con-2/remdesivir) against Mpro/3CLpro, PLpro and ACE2 of COVID-19

Receptor/target	Ligand/compound	Conventional hydrogen bonds with corresponding bond length	Amino acid residues involved in hydrophobic interaction
Mpro/3CLpro (6LU7)	C3/Luteolin	O4––OG1(Thr25) (2.88 Å) O3––(His41) (2.69 Å) O3––O(Cys44) (3.01 Å) O4––O(Cys44) (2.92 Å) O1––N(Gly143) (2.86 Å) O6––N(Glu166) (2.93 Å)	Thr45, Met49, Asn142, Cys145, Met165 and Asp187 (*N* = 6)
	C43/Abyssinone II	O4––O(Leu141) (2.78 Å) O4––OG(Ser144) (3.07 Å)	Thr25, Thr26, Leu27, Met49, Asn142, Gly143, Cys145, His163, His164, Met165 and Glu166 (*N* = 11)
	Con-2/Remdesivir	O8––N(Gly143) (3.11 Å) N6––O(Glu166) (2.83 Å) N6––NE2(Gln189) (3.33 Å)	Thr24, Thr25, Thr26, Leu27, His41, Cys44, Thr45, Met49, Asn142, Cys145, His164 and Met165 (*N* = 12)
PLpro (6WX4)	C3/Luteolin	O5––OE1(Glu161) (3.18 Å)	Lys157, Leu162, Gly163, Asp164, Arg166, Tyr264, Tyr268, Gln269 and Tyr273 (*N* = 9)
	C43/Abyssinone II	O3––OE1(Glu161) (2.85 Å)	Gly160, Leu162, Gly163, Asp164, Tyr264 and Tyr268 (*N* = 6)
	Con-2/Remdesivir	N6––OH(Tyr112) (3.18 Å) N3––NE(Arg166) (3.28 Å) N3––NH2(Arg166) (3.1 Å) O7––OE2(Glu167) (2.92 Å) O8––OE2(Glu167) (2.71 Å)	Leu162, Gly163, Asp164, Met208, Ser245, Ala246, Pro247, Tyr264, Tyr268, Gl269 and Cys270 (*N* = 11)
ACE2 (1R4L)	C3/Luteolin	O3––O(Gly250) (3.21 Å) O3––OG1(Thr258) (3.08 Å) O3––ND2(Asn259) (2.8 Å) O5––OE2(Glu388) (3.07 Å)	Ala135, Asp251, Phe256, Asp349, Thr353, His356, Glu357 and Thr427 (*N* = 8)
	C43/Abyssinone II	O4––OD1(Asp350) (3.05 Å) O3––OE2(Glu388) (2.77 Å)	Glu127, Phe156, Trp253, His327, Pro328, Asp349, Leu352, His356, Thr363, Thr427 and Tyr497 (*N* = 11)
	Con-2/Remdesivir	O7––OE2(Glu127) (2.93 Å) N1––ND2(Asn131) (3.11 Å) O8––ND1(His327) (3.26 Å) O1––OG1(Thr353) (3.03 Å)	Asp251, Met252, Trp253, Arg255, Phe256, Thr258, Pro328, Lys345, Asp349, Asp350, Leu352, His356, Glu388, Ser391, Thr427 and Tyr497 (*N* = 16)

### MD simulation data analysis

**Fig. 6:**
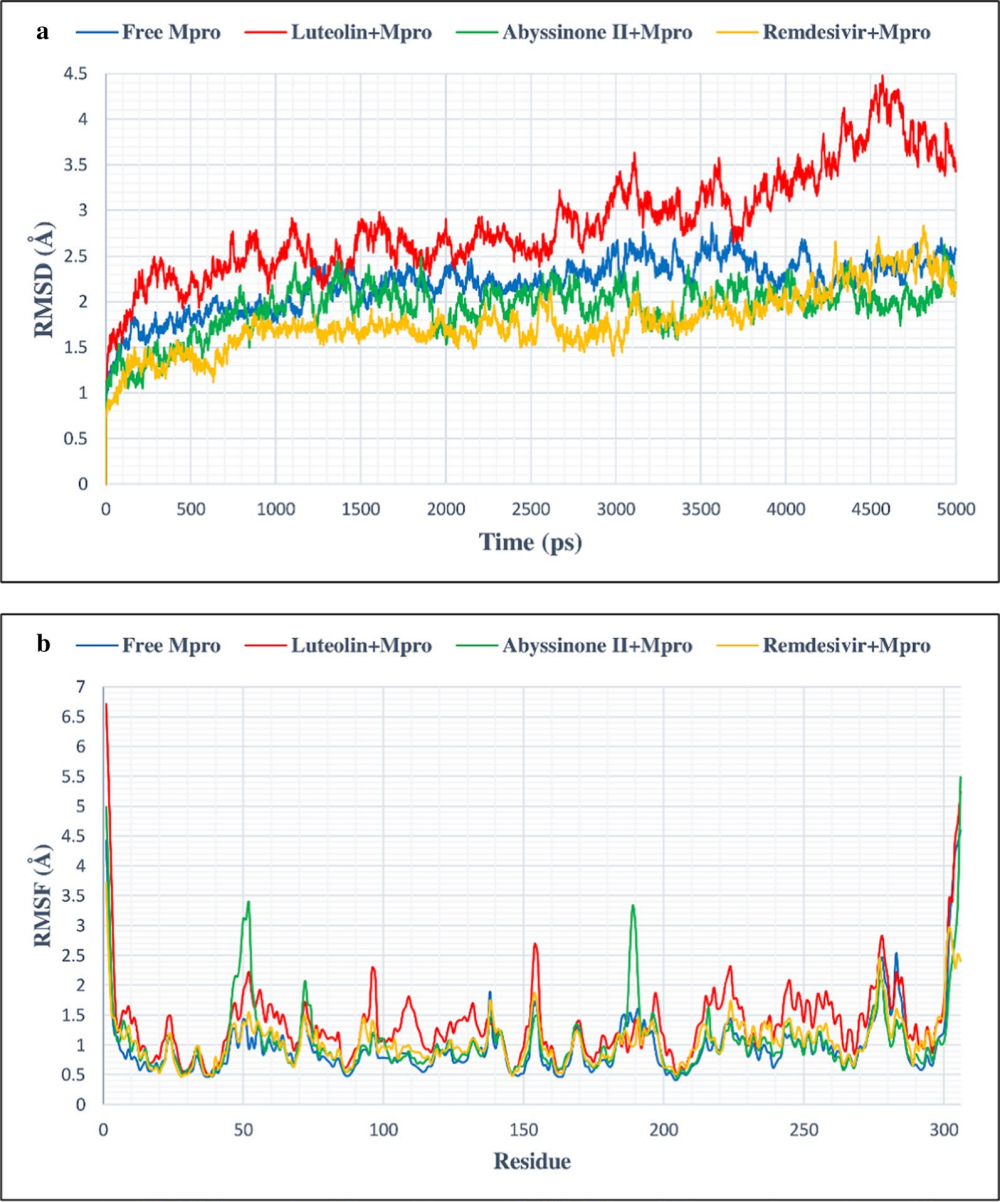
**a** RMSD plot of 5 ns MD simulation for free and bound Mpro/3CLpro complexed with C3/luteolin, C43/abyssinone II and Con-2/remdesivir, **b** RMSF plot of free and bound Mpro/3CLpro complexed with C3/luteolin, C43/abyssinone II and Con-2/remdesivir

**Fig. 7:**
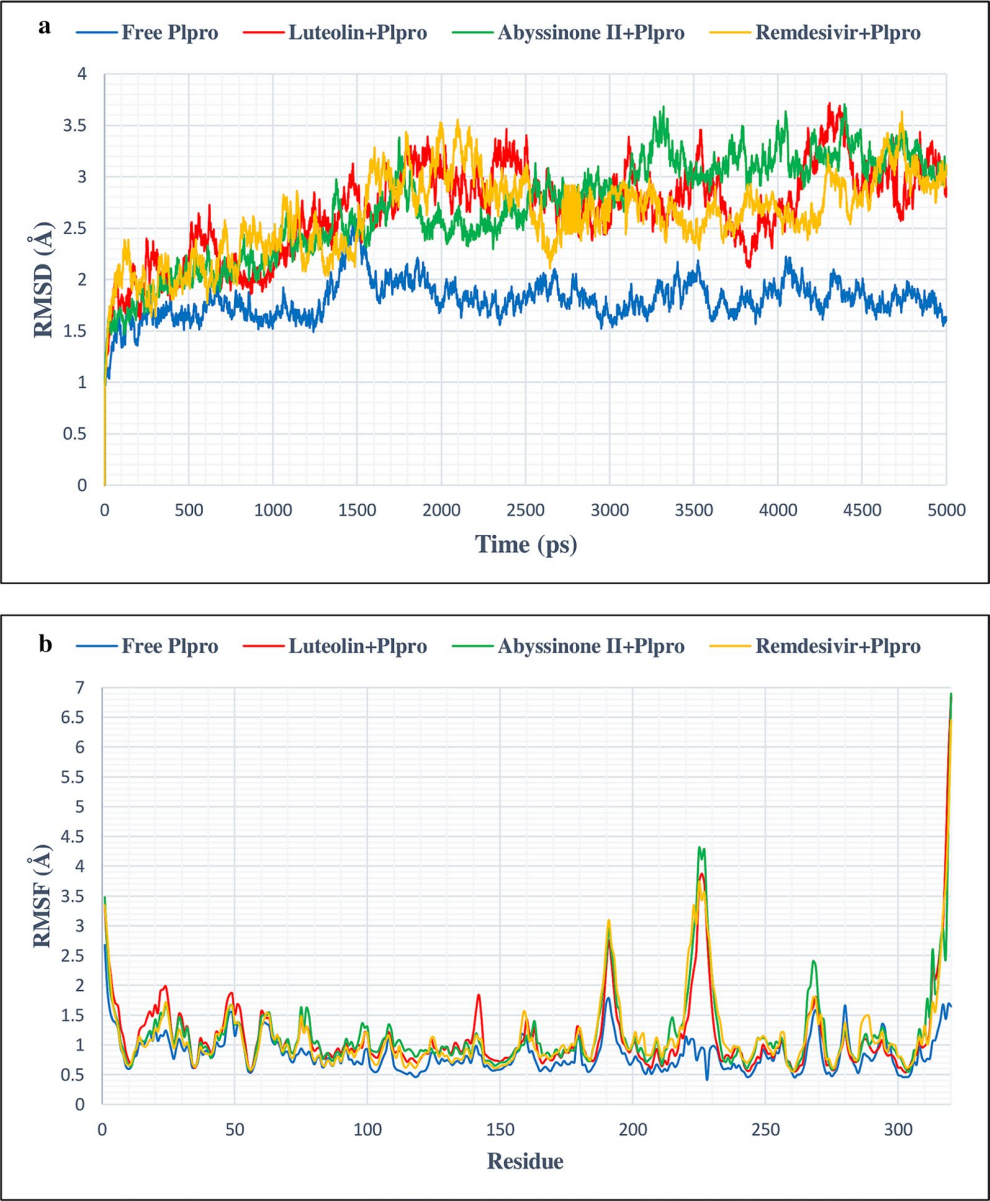
**a** RMSD plot of 5 ns MD simulation for free and bound PLpro complexed with C3/luteolin, C43/abyssinone II and Con-2/remdesivir, **b** RMSF plot of free and bound PLpro complexed with C3/luteolin, C43/abyssinone II and Con-2/remdesivir

**Fig. 8:**
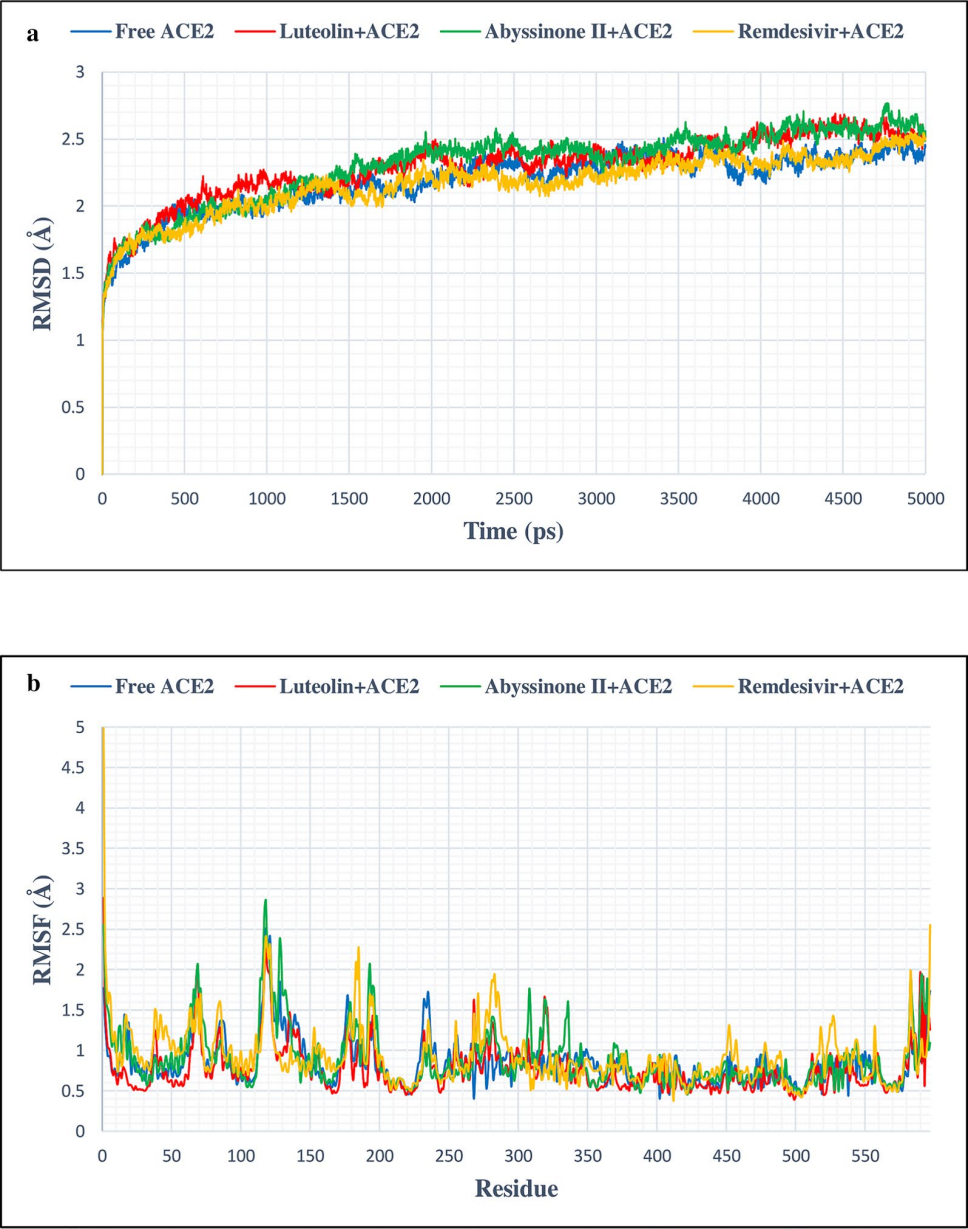
**a** RMSD plot of 5 ns MD simulation for free and bound ACE2 complexed with C3/luteolin, C43/abyssinone II and Con-2/remdesivir, **b** RMSF plot of free and bound ACE2 complexed with C3/luteolin, C43/abyssinone II and Con-2/remdesivir

In this study, MD simulation was done to corroborate the stability of protein–ligand complexes. This was further validated by evaluating of RMSD and RMSF values. Free Mpro/3CLpro protein showed stable RMSD from 1300 picosecond (ps) to 5000 ps timeline averaging RMSD value of 2.3 Å (Fig. 6a). This figure clearly demonstrates that C3/luteolin and Mpro/3CLpro complex displayed stable RMSD value averaging 2.9 Å from 300 to 3900 ps, which then showed an increase in RMSD value till the finish of the simulation. Simulation of C43/abyssinone II and Mpro/3CLpro complex confirmed stable conformation displaying average RMSD value of 2 Å between 1100 and 3100 ps, following a marginal deviation till the end. On the other hand, Con-2/remdisivir and Mpro/3CLpro complex showed steady RMSD value averaging 1.88 Å between 900 and 3100 ps followed by a slight deviation from the straight line till the termination of the run (Fig. 6b). Figure 6b depicts an oscillation of RMSF value between 0.6 and 2.1 Å on average, suggesting that tested ligands were in close proximity with the binding site of Mpro/3CLpro. From the simulation study of PLpro and its associated compounds (Fig. 7a), it was seen that free PLpro displayed steady RMSD value averaging 1.8 Å from 1600 to 5000 ps; C3/luteolin and PLpro complex also showed stable RMSD of 2.9 Å on average from 1600 ps to till the end of simulation. Likewise, C43/abyssinone II and PLpro complex gained stability after 1600 ps and remained stable throughout the simulation with an average RMSD of 2.97 Å; the other complex Con-2/remdisivir and PLpro showed steadiness of RMSD value with an average of 2.8 Å from 1900 ps to the finish of the simulation. Even though the complex conformation displayed steadiness after specific points of time, the ligand bindings affect the RMSD of protein backbone. Figure 7b displays the RMSF calculation of PLpro complex with the tested ligands, which showed significant fluctuation occurred between 225 and 235 residues, signifying the binding stability of the ligands associated with their respective protein with the best pose conformation. RMSD fluctuation of free ACE2 and its associated ligands was visualized in Fig. 8a. After 1500 ps, free ACE2 as well as its linked compounds C3/luteolin, C43/abyssinone II and Con-2/remdisivir attained stable RMSD value averaging 2.3 Å, 2.4 Å, 2.46 Å and 2.2 Å, respectively throughout the run. During the course of MD simulation, the RMSF changes were minor except the oscillation of RMSF values between 110–140 and 180–200 amino acid residues (Fig. 8b).

### Prediction of molecular targets within *H. sapiens*

**Fig. 9:**
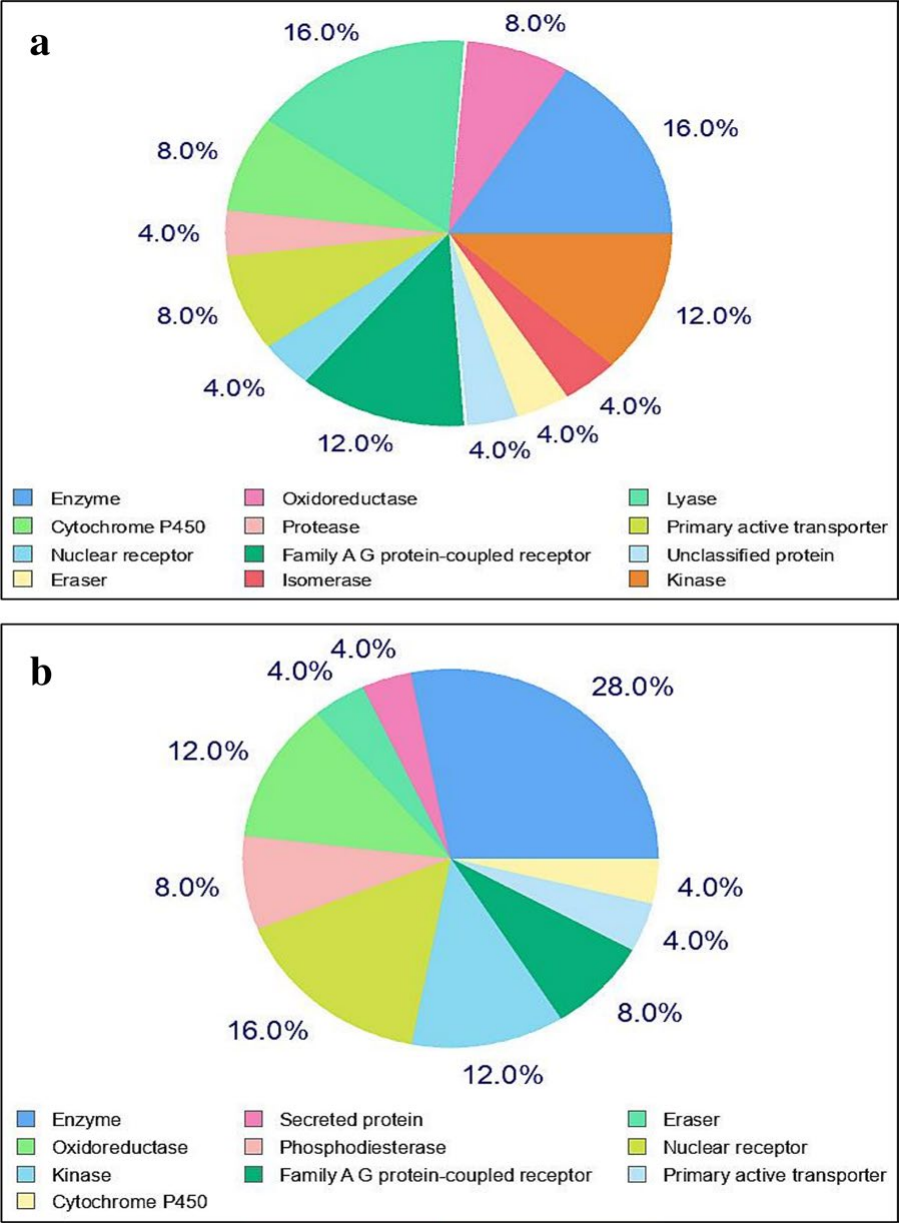
Top 25 classes of molecular targets within *H. sapiens* predicted for **a** C3/luteolin and **b** C43/abyssinone II

The molecular targets for C3/luteolin and C43/abyssinone II within human being were analyzed using SwissTargetPrediction web server under the hood of SIB (Swiss Institute of Bioinformatics). Among the top 25 classes of target prediction, it was found that enzymes, cytochrome P450, nuclear receptors, kinase, oxidoreductases, AG protein coupled receptors and primary active transporters might act as common molecular targets for C3/luteolin and C43/abyssinone II in human (Fig. 9). However, the probability scores for the selected flavonoids to hit different molecular targets within *H. sapiens* were found to be negligible (i.e., 0.1289–0.1047 and 0.3320–0.1120 for C3/luteolin and C43/abyssinone II, respectively) (Additional file 1: Table S2.1 and Table S2.2). Therefore, it can be concluded that the two tiny flavonoids are highly capable of getting entry into the active sites of Mpro or 3CLpro, PLpro and ACE2.

## Conclusion

The life-threatening COVID-19 continues its surge across the globe and has already caused more than 1.1 million deaths. At present, no effective drugs or vaccines are available against this deadly pandemic. Therefore, the design and development of most unique as well as potent anti-SARS-CoV-2 agent is terribly important in this hour of need. In this study, structure-based targeting of Mpro/3CLpro, Plpro and ACE2 was done with a small library of different flavonoids having antiviral activity to detect most potential flavonoid molecules against COVID-19. Two potent flavonoids, i.e., C3/luteolin and C43/abyssinone II, exhibited the highest binding affinity (Δ*G*^0^) and lowest inhibition constant (Ki) compared to other compounds (reference molecules, flavonoids and control drugs) against all the 3 potent targets of COVID-19. The outcome of MD trajectory analysis revealed that the studied protein–ligand complexes were structurally stable throughout the MD simulation. Therefore, C3/luteolin and C43/abyssinone II having harmless ADMET features interact to their targets more effectively and can act as most potent antiviral agents against SARS-CoV-2. For the innovation and development of novel anti-COVID-19 compounds, findings of this research demand further authentication in vivo and in vitro.

## Supplementary information

**Additional file 1.** Supplementary information of this article including Fig. S1, Fig. S2, Table S1, Table S2.1 and Table S2.2 are provided at the end of this manuscript.

## Data Availability

All the data generated or analyzed during this study are included in this published article.

## Abbreviations

COVID-19: Coronavirus disease-2019
SARS-CoV-2: Severe Acute Respiratory Syndrome Coronavirus 2
WHO: World Health Organization
2019-nCoV: 2019-Novel coronavirus
ICCSG: International Committee of Coronavirus Study Group
ARDS: Acute Respiratory Distress Syndrome
RNA: Ribonucleic Acid
MERS-CoV: Middle East Respiratory Syndrome Coronavirus
S protein: Spike protein
RdRp: RNA-dependent RNA polymerase
Mpro: Main protease
3CLpro: 3 Chymotrypsin-like protease
PLpro: Papain-like protease
ACE2: Angiotensin converting enzyme-2
TMPRSS2: Transmembrane protease serine 2
FDA: Food and Drug Administration
VLS: Virtual ligand screening
HSV: Herpes simplex virus
HIV: Human immunodeficiency virus
HCV: Hepatitis C virus
SDF: Spatial Data File
PDB: Protein Data Bank
3D: 3 Dimensional
ADMET: Absorption, Distribution, Metabolism, Excretion and Toxicity
SMILES: Simplified Molecular Input Line Entry System
RMSD: Root mean square deviation
ΔG^0^: Gibbs free energy
Ki: Inhibition constant
MD: Molecular dynamics
NAMD: Nanoscalable molecular dynamics
VMD: Visual molecular dynamics
PSF: Protein Structure File
ns: Nanosecond
ps: Picosecond
fs: Femtosecond
RMSF: Root mean square fluctuation
MW: Molecular weight
HBD: Hydrogen bond donor
HBA: Hydrogen bond acceptor
TPSA: Topological polar surface area
HIA: Human intestinal absorption
BBB: Blood brain barrier
nM: Nanomolar
Kcal: Kilo calorie
EBV: Epstein Barr Virus
SIB: Swiss Institute of Bioinformatics

## References

[CR1] Daina A, Michielin O, Zoete V (2019). SwissTargetPrediction: updated data and new features for efficient prediction of protein targets of small molecules. Nucleic Acids Res.

[CR2] Devaux C, Rolain J, Colson P, Raoult D (2020). New insights on the antiviral effects of chloroquine against coronavirus: what to expect for COVID-19?. Int J Antimicrob Agents.

[CR3] Du L, He Y, Zhou Y, Liu S, Zheng B, Jiang S (2009). The spike protein of SARS-CoV-a target for vaccine and therapeutic development. Nat Rev Microbiol.

[CR4] Elmezayen A, Al-Obaidi A, Şahin A, Yelekçi K (2020). Drug repurposing for coronavirus (COVID-19): in silico screening of known drugs against coronavirus 3CL hydrolase and protease enzymes. J Biomol Struct Dyn.

[CR5] Enayatkhani M, Hasaniazad M, Faezi S, Gouklani H, Davoodian P, Ahmadi N (2020). Reverse vaccinology approach to design a novel multi-epitope vaccine candidate against COVID-19: an in silico study. J Biomol Struct Dyn.

[CR6] Enmozhi S, Raja K, Sebastine I, Joseph J (2020). Andrographolide as a potential inhibitor of SARS-CoV-2 main protease: an in silico approach. J Biomol Struct Dyn.

[CR7] Ghersi D, Sanchez R (2009). Improving accuracy and efficiency of blind protein-ligand docking by focusing on predicted binding sites. Proteins.

[CR8] Guan L, Yang H, Cai Y (2018). ADMET-score—a comprehensive scoring function for evaluation of chemical drug-likeness. Medchemcomm.

[CR9] Guan W, Ni Z, Hu Y, Liang W, Ou C, He J (2020). Clinical characteristics of coronavirus disease 2019 in China. N Engl J Med.

[CR10] Guo Y, Cao Q, Hong Z, Tan Y, Chen S, Jin H (2020). The origin, transmission and clinical therapies on coronavirus disease 2019 (COVID-19) outbreak—an update on the status. Mil Med Res.

[CR11] Gurung A, Ali M, Lee J, Farah M, Al-Anazi K (2020). Unravelling lead antiviral phytochemicals for the inhibition of SARS-CoV-2 Mpro enzyme through in silico approach. Life Sci.

[CR12] Hanwell M, Curtis D, Lonie D, Vandermeersch T, Zurek E, Hutchison G (2012). Avogadro: an advanced semantic chemical editor, visualization, and analysis platform. J Cheminform.

[CR13] Hilgenfeld R (2014). From SARS to MERS: crystallographic studies on coronaviral proteases enable antiviral drug design. FEBS J.

[CR14] Hoffmann M, Kleine-Weber H, Schroeder S, Krüger N, Herrler T, Erichsen S (2020). SARS-CoV-2 cell entry depends on ACE2 and TMPRSS2 and is blocked by a clinically proven protease inhibitor. Cell.

[CR15] Humphrey W, Dalke A, Schulten K (1996). VMD: visual molecular dynamics. J Mol Graph.

[CR16] Jo S, Kim T, Iyer VG, Im W (2008). CHARMM-GUI: a web-based graphical user interface for CHARMM. J Comput Chem.

[CR17] Johansson M, Zoete V, Michielin O, Guex N (2012). Defining and searching for structural motifs using DeepView/Swiss-PdbViewer. BMC Bioinform.

[CR18] Joshi T, Joshi T, Sharma P, Mathpal S, Pundir H, Bhatt V (2020). In silico screening of natural compounds against COVID-19 by targeting Mpro and ACE2 using molecular docking. Eur Rev Med Pharmacol Sci.

[CR19] Kitchen D, Decornez H, Furr J, Bajorath J (2004). Docking and scoring in virtual screening for drug discovery: methods and applications. Nat Rev Drug Discov.

[CR20] Kuba K, Imai Y, Rao S, Gao H, Guo F, Guan B (2005). A crucial role of angiotensin converting enzyme 2 (ACE2) in SARS coronavirus–induced lung injury. Nat Med.

[CR21] Liu C, Zhou Q, Li Y, Garner L, Watkins S, Carter L (2020). Research and development on therapeutic agents and vaccines for COVID-19 and related human coronavirus diseases. ACS Cent Sci.

[CR22] MacKerell AD, Bashford D, Bellott M (1998). All-atom empirical potential for molecular modeling and dynamics studies of proteins. J Phys Chem B.

[CR23] Mehta N, Mazer-Amirshahi M, Alkindi N, Pourmand A (2020). Pharmacotherapy in COVID-19; a narrative review for emergency providers. Am J Emerg Med.

[CR24] Meng X, Zhang H, Mezei M, Cui M (2011). Molecular docking: a powerful approach for structure-based drug discovery. Curr Comput Aided Drug Des.

[CR25] Meyerowitz EA, Vannier AGL, Friesen MGN (2020). Rethinking the role of hydroxychloroquine in the treatment of COVID-19. FASEB J.

[CR26] Mohammadi Pour P, Fakhri S, Asgary S, Farzaei M, Echeverría J (2019). The signaling pathways, and therapeutic targets of antiviral agents: focusing on the antiviral approaches and clinical perspectives of anthocyanins in the management of viral diseases. Front Pharmacol.

[CR27] Molyneux R, Lee S, Gardner D, Panter K, James L (2007). Phytochemicals: the good, the bad and the ugly?. Phytochemistry.

[CR28] Newman D, Cragg G (2007). Natural products as sources of new drugs over the last 25 years. J Nat Prod.

[CR29] Nosrati M, Hajizade A, Nazarian S, Amani J, Namvar Vansofla A, Tarverdizadeh Y (2019). Designing a multi-epitope vaccine for cross-protection against Shigella spp: an immunoinformatics and structural vaccinology study. Mol Immunol.

[CR30] Notka F, Meier G, Wagner R (2004). Concerted inhibitory activities of on HIV replication in vitro and ex vivo. Antiviral Res.

[CR31] Pettersen E, Goddard T, Huang C, Couch G, Greenblatt D, Meng E (2004). UCSF Chimera- a visualization system for exploratory research and analysis. J Comput Chem.

[CR32] Phillips JC, Braun R, Wang W (2005). Scalable molecular dynamics with NAMD. J Comput Chem.

[CR33] Rahman A, Ali MT, Shawan MM, Sarwar MG, Khan MA, Halim MA (2016). Halogen-directed drug design for Alzheimer's disease: a combined density functional and molecular docking study. Springerplus.

[CR34] Ramírez D, Caballero J (2018). Is it reliable to take the molecular docking top scoring position as the best solution without considering available structural data?. Molecules.

[CR35] Rasool N, Ashraf A, Waseem M, Hussain W, Mahmood S (2018). Computational exploration of antiviral activity of phytochemicals against NS2B/NS3 proteases from dengue virus. Turk J Biochem.

[CR36] Rehman S, Ashfaq U, Riaz S, Javed T, Riazuddin S (2011). Antiviral activity of *Acacia nilotica* against Hepatitis C Virus in liver infected cells. Virol J.

[CR37] Rut W, Lv Z, Zmudzinski M, Patchett S, Nayak D, Snipas S et al (2020) Activity profiling and structures of inhibitor-bound SARS-CoV-2-PLpro protease provides a framework for anti-COVID-19 drug design. bioRxiv 6(42). 10.1101/2020.04.29.068890. 10.1126/sciadv.abd4596 PMC7567588 33067239

[CR38] Samofalova D, Karpov P, Raevsky A, Blume Y (2017). Protein phosphatases potentially associated with regulation of microtubules, their spatial structure reconstruction and analysis. Cell Biol Int.

[CR39] Shah B, Modi P, Sagar S (2020). In silico studies on therapeutic agents for COVID-19: drug repurposing approach. Life Sci.

[CR40] Snøve O, Holen T (2004). Many commonly used siRNAs risk off-target activity. Biochem Biophys Res Commun.

[CR41] Song JJ, Smith SK, Hannon GJ, Joshua-Tor L (2004). Crystal structure of Argonaute and its implications for RISC slicer activity. Science.

[CR42] Towler P, Staker B, Prasad S, Menon S, Tang J, Parsons T (2004). ACE2 X-ray structures reveal a large hinge-bending motion important for inhibitor binding and catalysis. J Biol Chem.

[CR43] Umar A, Uzairu A, Shallangwa G, Uba S (2020). Docking-based strategy to design novel flavone-based arylamides as potent V600E-BRAF inhibitors with prediction of their drug-likeness and ADMET properties. Bull Natl Res Cent.

[CR44] Uno Y (2020). Camostat mesilate therapy for COVID-19. Intern Emerg Med.

[CR45] World Health Organization (WHO) (2020) Coronavirus disease (COVID-19) pandemic. https://www.who.int/emergencies/diseases/novel-coronavirus-2019. Accessed 13 Nov 2020

[CR46] Zakaryan H, Arabyan E, Oo A, Zandi K (2017). Flavonoids: promising natural compounds against viral infections. Arch Virol.

