# Supplementary material for: Luteolin and abyssinone II as potential inhibitors of SARS-CoV-2: an in silico molecular modeling approach in battling the COVID-19 outbreak
Source: Bull Natl Res Cent. 2021 Jan 20;45(1):27. doi: 10.1186/s42269-020-00479-6 (PMC7816153; doi:10.1186/s42269-020-00479-6)
Supplement: Supplementary file 1 — Additional file 1. Supplementary information of this article including Fig. S1, Fig. S2, Table S1, Table S2.1 and Table S2.2 are provided at the end of this manuscript. [file 42269_2020_479_MOESM1_ESM.docx]

**Luteolin and abyssinone II as potential inhibitors of SARS-CoV-2: an *in silico* molecular modeling approach in battling the COVID-19 outbreak**

Mohammad Mahfuz Ali Khan Shawan*, Sajal Kumar Halder and Md. Ashraful Hasan*

Department of Biochemistry and Molecular Biology, Jahangirnagar University, Savar, Dhaka-1342, Bangladesh.

**(SUPPLEMENTARY INFORMATION)**

*Corresponding author

**Mohammad Mahfuz Ali Khan Shawan, M.Sc.**

Assistant Professor

Department of Biochemistry and Molecular Biology,

Jahangirnagar University, Savar, Dhaka 1342, Bangladesh.

Email: [mahfuz_026shawan@juniv.edu](mailto:mahfuz_026shawan@juniv.edu); mahfuz_026shawan@yahoo.com

Tel: +8801714451833; +8801797547130

**Md. Ashraful Hasan, Ph.D.**

Associate Professor

Department of Biochemistry and Molecular Biology,

Jahangirnagar University, Savar, Dhaka 1342, Bangladesh.

Email: ashrafulhasan@juniv.edu

Tel: +8801933123455

**Supplementary Figure captions**

**Fig. S1** 2D Structures of selected antiviral flavonoids (phytochemials) and drugs (used as control) for docking against Mpro/3CLpro, PLpro and ACE2 of COVID-19

**Fig. S2** 3D structures (cartoon view) of **a)** SARS-CoV-2 Mpro/3CLpro (magenta) bound with inhibitor N3 (light green), **b)** SARS-CoV-2 PLpro (cyan) bound with inhibitor VIR251 (red) and **c)** Human ACE2 (red) bound with inhibitor XX5 (deep blue)

**Supplementary Table captions**

**Table S1** The summary of adopted flavonoids and control drugs used for virtual screening against Mpro/3CLpro, PLpro and ACE2 of COVID-19. Here C1-C10 belongs to flavone, C11-C21 belongs to flavonol, C22-C23 belongs to chalcone, C24-C27 belongs to flavan, C28-C34 belongs to isoflavone, C35-C39 belongs to anthocyanidin and C40-C43 belongs to flavanone class of flavonoids

**Table S2.1** Molecular target sites for C3/luteolin within *H. sapiens* predicted by SwissTargetPrediction web server

**Table S2.2** Molecular target sites for C43/abyssinone II within *H. sapiens* predicted by SwissTargetPrediction web server


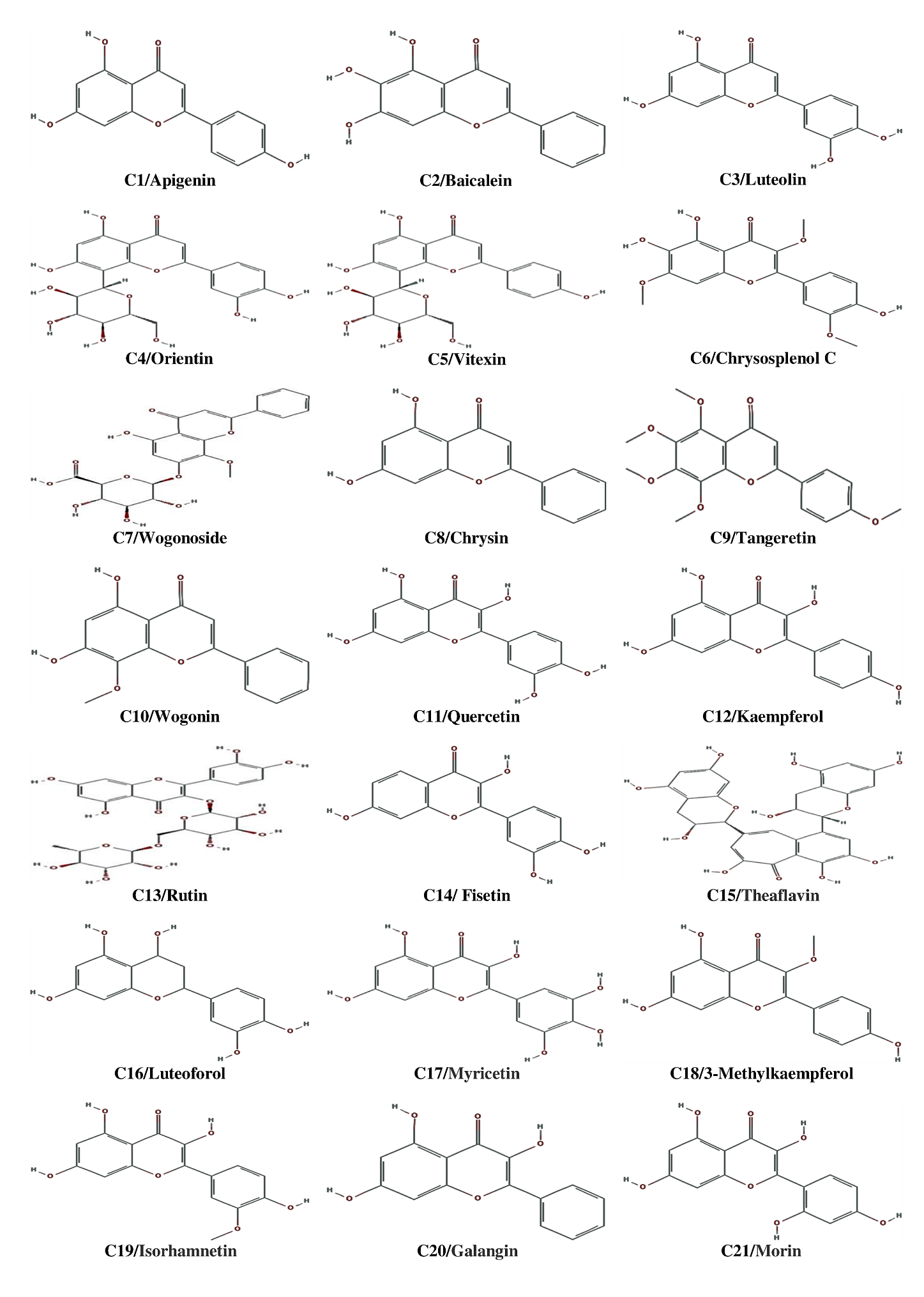
**Fig. S1**

**
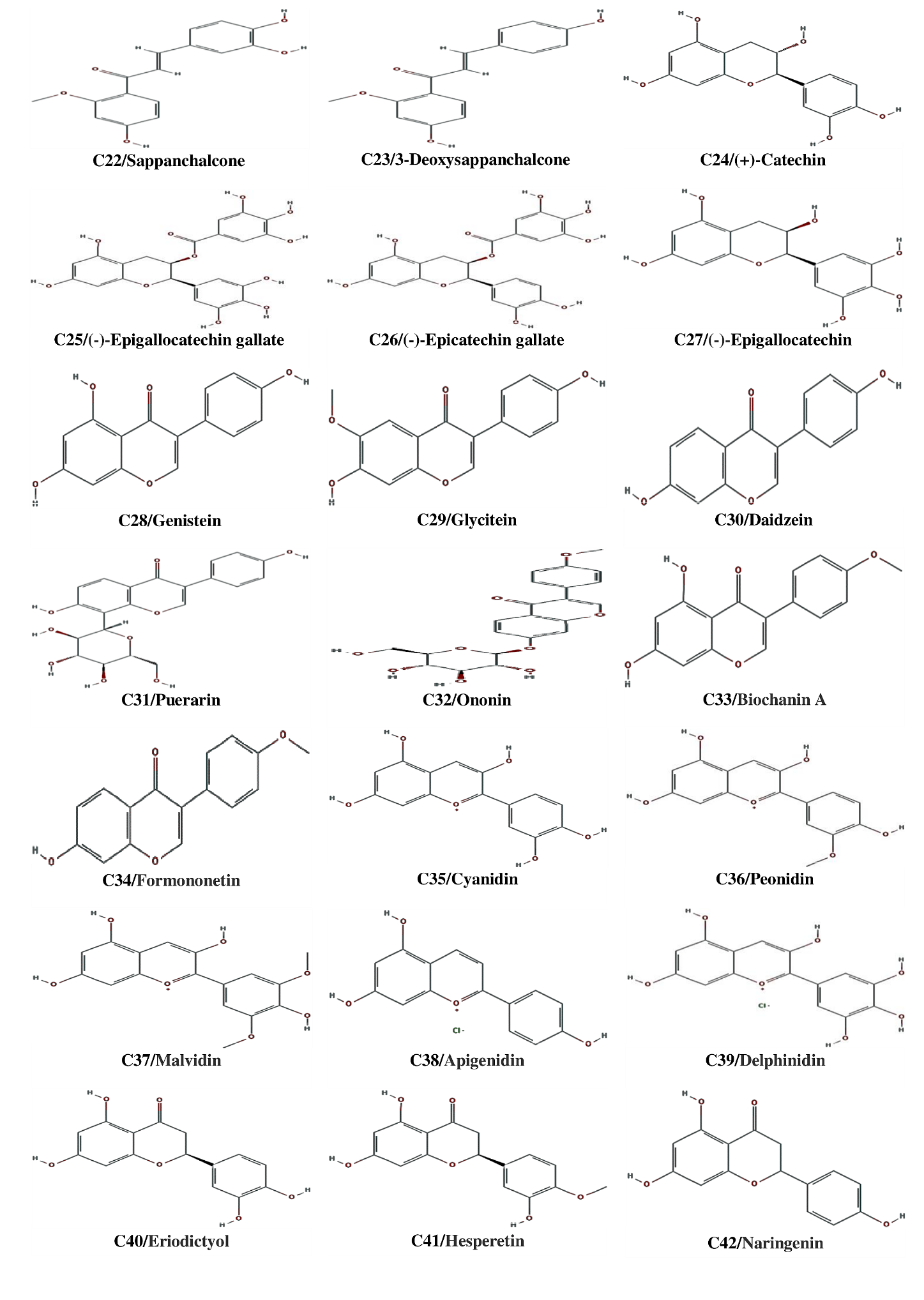
Fig. S1 (Continued_1)**

**Fig. S1 (Continued_2)**


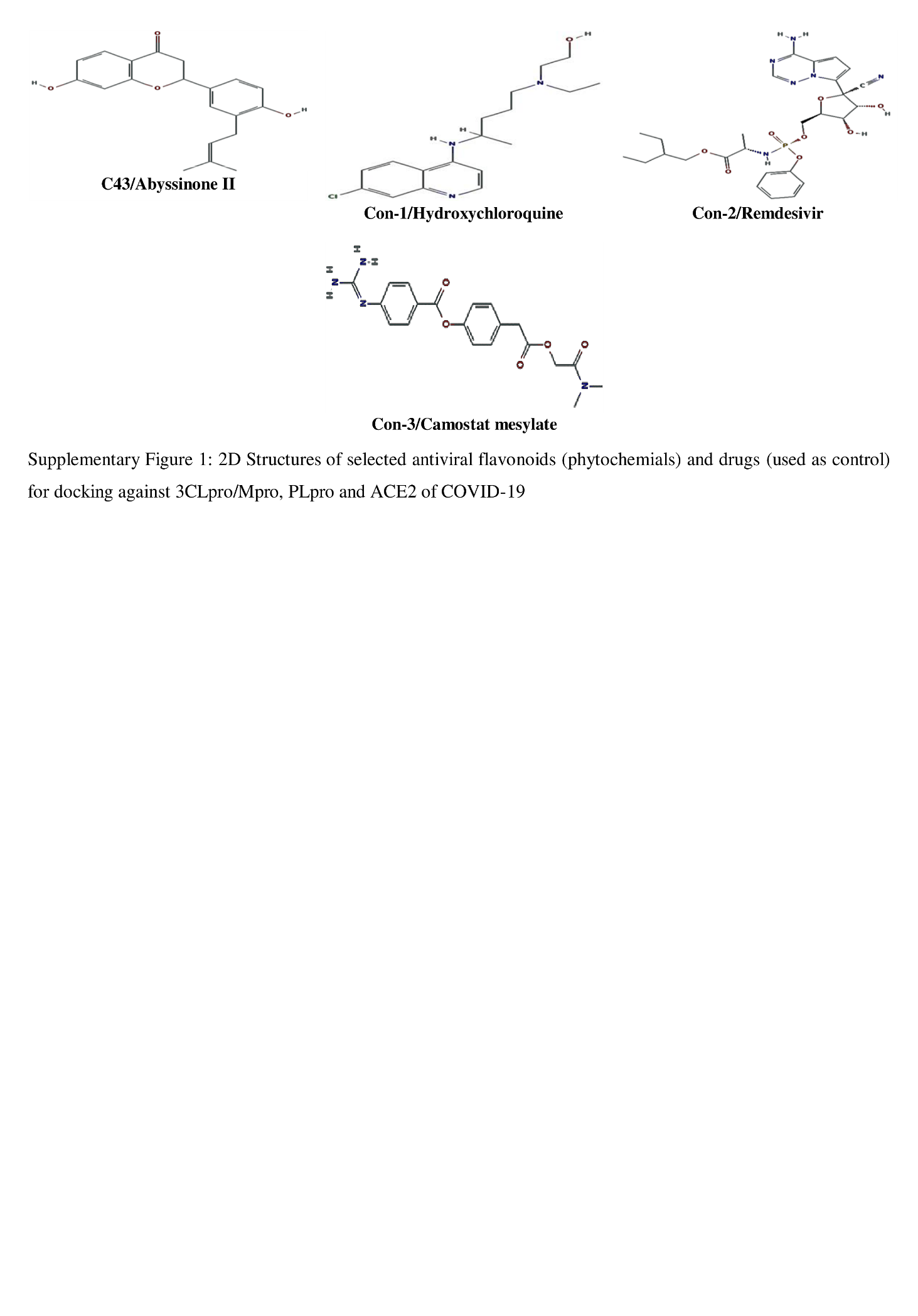


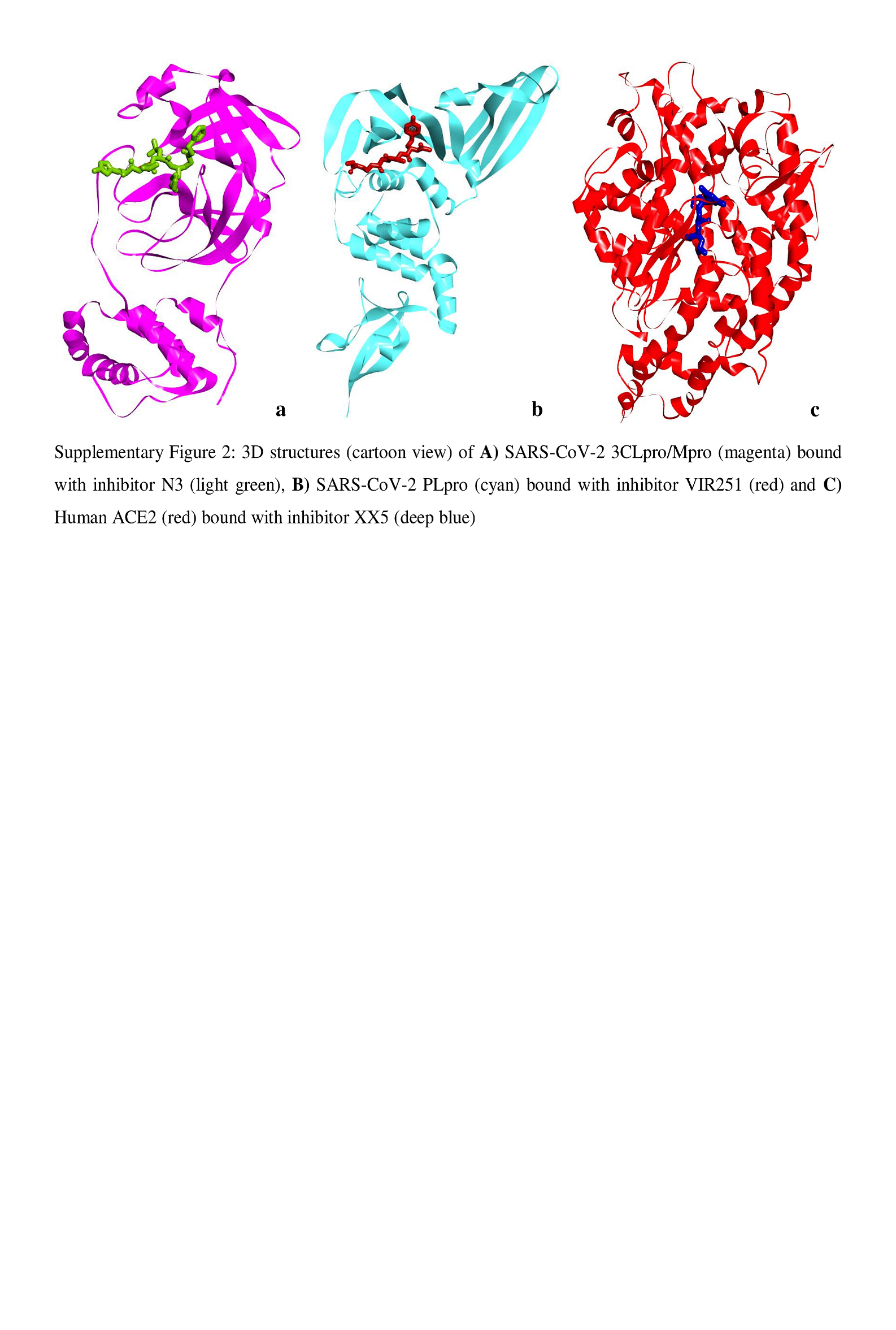
**Fig. S2**

**Table S1**

| **Name/Flavonoids** | **PubChem CID** | **Canonical SMILES** | **Plant** | **Antiviral activity against** | **Mechanism of action** | **Ref.** |
| --- | --- | --- | --- | --- | --- | --- |
| C1/Apigenin | 5280443 | C1=CC(=CC=C1C2=CC(=O)C3=C(C=C(C=C3O2)O)O)O | *Matricaria chamomilla* and *Petroselinum crispum* | HSV-1, HSV-2, HCV, Influenza virus and Enterovirus 71 (EV71) | Inhibition of early and late phase of viral replication | [Wang et al. 2019] |
| C2/Baicalein | 5281605 | C1=CC=C(C=C1)C2=CC(=O)C3=C(O2)C=C(C(=C3O)O)O | *Scutellaria baicalensis* and *Scutellaria lateriflora* | HCMV, DENV, Influenza virus and Japanese encephalitis virus (JEV) | Inhibition of viral replication, transcription and translation | [Zakaryan et al. 2017] |
| C3/Luteolin | 5280445 | C1=CC(=C(C=C1C2=CC(=O)C3=C(C=C(C=C3O2)O)O)O)O | *Ocimum basilicum*, *Spinacia*  *oleracea* and *Capsicum annuum* | HIV-1, EBV, EV71, SARS-CoV, Influenza virus and JEV | Disruption of viral RNA replication, inhibition of viral reactivation by blocking LTR transactivation | [Zakaryan et al. 2017] |
| C4/Orientin | 5281675 | C1=CC(=C(C=C1C2=CC(=O)C3=C(O2)C(=C(C=C3O)O)C4C(C(C(C(O4)CO)O)O)O)O)O | Ocimum sanctum*,* *Trollius chinensis* and Jatropha gossypifolia | Influenza and Parainfluenza type 3 virus | Inhibition of neuraminidase activity and acceleration of TNF-α expression | [Xu et al. 2020] |
| C5/Vitexin | 5280441 | C1=CC(=CC=C1C2=CC(=O)C3=C(O2)C(=C(C=C3O)O)C4C(C(C(C(O4)CO)O)O)O)O | *Trollius chinensis*, *Erythrina speciose* and *Crataegus pinnatifida* | Rotavirus, HSV-1, Parainfluenza type 3 virus and HIV | Yet not detected | [He et al. 2016] |
| C6/Chrysosplenol C | 189065 | COC1=C(C=CC(=C1)C2=C(C(=O)C3=C(C(=C(C=C3O2)OC)O)O)OC)O | *Pterocaulon sphacelatum* and *Chrysosplenium tosaense* | HIV and Rhinovirus | Inhibition of RNA synthesis | [Semple et al. 1999] |
| C7/Wogonoside | [3084961](https://pubchem.ncbi.nlm.nih.gov/compound/3084961) | COC1=C(C=C(C2=C1OC(=CC2=O)C3=CC=CC=C3)O)OC4C(C(C(C(O4)C(=O)O)O)O)O | *Scutellaria baicalensis* | SARS beta coronavirus | Interfere with the activation of NLRP3 inflammasome | [McKee et al. 2020] |
| C8/Chrysin | 5281607 | C1=CC=C(C=C1)C2=CC(=O)C3=C(C=C(C=C3O2)O)O | *Passiflora caerulea*  and *Matricaria*  *chamomilla* | HSV-1, HIV, Coxsackievirus B3 and EV71 | Interfere with viral activation by suppressing 3CL protease | [Song et al. 2015] |
| C9/Tangeretin | 68077 | COC1=CC=C(C=C1)C2=CC(=O)C3=C(O2)C(=C(C(=C3OC)OC)OC)OC | Citrus reticulate | *Human respiratory syncytial virus (HRSV)* | Inhibition of  intracellular replication | [Nothias-Scaglia et al. 2014] |
| C10/Wogonin | 5281703 | COC1=C(C=C(C2=C1OC(=CC2=O)C3=CC=CC=C3)O)O | *Scutellaria*  *baicalensis* | HBV, Influenza virus A and B | Upregulation of interferon (IFN)-induced antiviral signaling and AMPK activation | [Seong et al. 2018] |
| C11/Quercetin | 5280343 | C1=CC(=C(C=C1C2=C(C(=O)C3=C(C=C(C=C3O2)O)O)O)O)O | *Raphanus raphanistrum*, *Foeniculum vulgare*, *Capsicum annuum* and  Embelia ribes | Rhinovirus, DENV-2, JEV, Adenovirus, HSV-1, HSV-2, Polio virus type-1, Influenza virus A and HRSV | Induction of type 1 IFN secretion causes the inhibition of viral replication, suppression of NF-kB activation and blocking of virus binding and penetration to the host cell | [Zakaryan et al. 2017] |
| C12/Kaempferol | 5280863 | C1=CC(=CC=C1C2=C(C(=O)C3=C(C=C(C=C3O2)O)O)O)O | *Dianella longifolia*, *Pterocaulus sphaedatum* and  *Psiadia dentata* | HCMV, HSV-1, HSV-2, HIV-1, Influenza virus and JEV | Inhibition of viral replication, protein synthesis and virus release | [Zakaryan et al. 2017] |
| C13/Rutin | 5280805 | CC1C(C(C(C(O1)OCC2C(C(C(C(O2)OC3=C(OC4=CC(=CC(=C4C3=O)O)O)C5=CC(=C(C=C5)O)O)O)O)O)O)O)O | *Fagopyrum tataricum*, *Rheum*  *rhabarbarum*, *Citrus aurantium* and *Citrus limon* | Parainfluenza type 3 virus, HIV-1 and EV71 | Inhibition of viral infection by suppressing the  activation of MEK1-ERK signal pathway and blocking viral entry and cell fusion | [Zakaryan et al. 2017] |
| C14/Fisetin | 5281614 | C1=CC(=C(C=C1C2=C(C(=O)C3=C(O2)C=C(C=C3)O)O)O)O | *Acacia* greggii, *Acacia berlandieri*, and *Fragaria*  *ananassa* | Chikungunya virus and Enterovirus A71 (EV A71) | Inhibition of the activity of 3C protease causes the decrease in the production of non-structural proteins and inhibition of viral infection | [Zakaryan et al. 2017] |
| C15/Theaflavin | 135403798 | C1C(C(OC2=CC(=CC(=C21)O)O)C3=CC4=C(C(=C(C=C4C5C(CC6=C(C=C(C=C6O5)O)O)O)O)O)C(=O)C(=C3)O)O | *Camellia sinensis* and *Eleutherococcus senticosus* | Rotavirus, Coronavirus, HSV-1 and HIV | Inhibition of RNA synthesis and exhibiting antiviral activity | [Lin et al. 1999; Yu et al. 2007] |
| C16/Luteoforol | 114505 | C1C(C2=C(C=C(C=C2OC1C3=CC(=C(C=C3)O)O)O)O)O | *Hypericum connatum* | HSV-1 | Inhibition of the cytopathic effect (CPE) and reducing the viral titer of HSV-1 | [Fritz et al. 2007] |
| C17/Myricetin | 5281672 | C1=C(C=C(C(=C1O)O)O)C2=C(C(=O)C3=C(C=C(C=C3O2)O)O)O | *Ipomoea batatas*,  *Petroselinum crispum* and *Camellia sinensis* | SARS-CoV, HBV, Influenza virus and HIV-1 | Inhibition of ATPase activity of viral helicase | [Zakaryan et al. 2017] |
| C18/  3-Methylkaempferol | 5280862 | COC1=C(OC2=CC(=CC(=C2C1=O)O)O)C3=CC=C(C=C3)O | *Zingiber zerumbet* | HSV-1 and Poliovirus | Inhibition of genomic RNA synthesis | [Andres et al. 2009] |
| C19/Isorhamnetin | 5281654 | COC1=C(C=CC(=C1)C2=C(C(=O)C3=C(C=C(C=C3O2)O)O)O)O | *Alphitonia philippinensis* | Influenza A virus, HSV-1 and EV71 | Inhibition of replication | [Jou et al. 2004] |
| C20/Galangin | 5281616 | C1=CC=C(C=C1)C2=C(C(=O)C3=C(C=C(C=C3O2)O)O)O | *Alpinia officinarum* and *Helichrysum aureonitens* | Coxsackie B virus type 1, HSV-1, HCV and HIV-1 | Inhibition of RNA synthesis | [Lin et al. 1999; Yu et al. 2007] |
| C21/Morin | 5281670 | C1=CC(=C(C=C1O)O)C2=C(C(=O)C3=C(C=C(C=C3O2)O)O)O | *Maclura pomifera*,  *Psidium guajava* and  *Maclura tinctoria* | HSV-2 | Inhibition of the early event in virus life cycle | [Malhotra et al. 1996] |
| C22/Sappanchalcone | 5319493 | COC1=C(C=CC(=C1)O)C(=O)C=CC2=CC(=C(C=C2)O)O | *Caesalpinia sappan* | Influenza virus | Yet not detected | [Liu et al. 2009] |
| C23/  3-Deoxysappanchalcone | 5319688 | COC1=C(C=CC(=C1)O)C(=O)C=CC2=CC=C(C=C2)O | *Caesalpinia sappan* | Influenza virus | Inhibition of viral genomic replication and transcription | [Yang et al. 2012] |
| C24/(+)-Catechin | 9064 | C1C(C(OC2=CC(=CC(=C21)O)O)C3=CC(=C(C=C3)O)O)O | *Theobroma cacao*, *Argania spinose* and *Camellia sinensis* | HSV-1, Adenovirus, Influenza virus, HIV-1, Rotavirus and HPV | Inhibition of viral replication by binding with different viral glycoproteins | [Zakaryan et al. 2017; Demeule et al. 2002] |
| C25/  (-)-Epigallocatechin gallate | 65064 | C1C(C(OC2=CC(=CC(=C21)O)O)C3=CC(=C(C(=C3)O)O)O)OC(=O)C4=CC(=C(C(=C4)O)O)O | *Camellia sinensis*, *Dianella longifolia*, *Malus pumila* and  *Psiadia dentata* | Influenza virus, HIV, Zika virus, HSV-1 and HCV | Interacts with viral structural proteins and prevents the recognition and binding of virus to cellular receptors | [Zakaryan et al. 2017; Li et al. 2020] |
| C26/  (-)-Epicatechin gallate | 107905 | C1C(C(OC2=CC(=CC(=C21)O)O)C3=CC(=C(C=C3)O)O)OC(=O)C4=CC(=C(C(=C4)O)O)O | *Camellia sinensis* | Influenza virus | Binds with haemagglutinin of influenza virus and preventing its adsorption | [Zakaryan et al. 2017] |
| C27/  (-)-Epigallocatechin | 72277 | C1C(C(OC2=CC(=CC(=C21)O)O)C3=CC(=C(C(=C3)O)O)O)O | *Camellia sinensis* | Influenza virus and HIV-1 | Reducing viral entry by clathrin mediated endocytosis | [Zakaryan et al. 2017] |
| C28/Genistein | 5280961 | C1=CC(=CC=C1C2=COC3=CC(=CC(=C3C2=O)O)O)O | *Vicia faba* and *Glycine max* | HSV-1, HSV-2, HIV, HCMV and Adenovirus | Inhibiting the function of immediate early proteins of viruses | [Evers et al. 2005] |
| C29/Glycitein | 5317750 | COC1=C(C=C2C(=C1)C(=O)C(=CO2)C3=CC=C(C=C3)O)O | *Vicia faba* and *Glycine max* | Influenza A and B virus | Yet not detected | [Nagai et al. 2019] |
| C30/Daidzein | 5281708 | C1=CC(=CC=C1C2=COC3=C(C2=O)C=CC(=C3)O)O | *Vicia faba* and *Glycine max* | JEV | Inhibiting the replication and protein expression of viruses | [Zakaryan et al. 2017] |
| C31/Puerarin | 5281807 | C1=CC(=CC=C1C2=COC3=C(C2=O)C=CC(=C3C4C(C(C(C(O4)CO)O)O)O)O)O | *Pueraria lobate*, *Vicia faba* and *Glycine max* | HRSV and HIV-1 | Inhibiting viral replication by blocking initial attachment of virus particles to the host cell surface | [Wu et al. 2020] |
| C32/Ononin | 442813 | COC1=CC=C(C=C1)C2=COC3=C(C2=O)C=CC(=C3)OC4C(C(C(C(O4)CO)O)O)O | *Vicia faba* and *Glycine max* | HBV | Activating interferon stimulated response element | [Yu et al. 2019] |
| C33/Biochanin A | 5280373 | COC1=CC=C(C=C1)C2=COC3=CC(=CC(=C3C2=O)O)O | *Trifolium pretense*, *Brassica oleracea* and *Medicago sativa* | Herpesvirus and Influenza A virus | Inhibition of viral antigen expression, nucleocapsid production and export of viral RNP complex | [Yu et al. 2019] |
| C34/Formononetin | 5280378 | COC1=CC=C(C=C1)C2=COC3=C(C2=O)C=CC(=C3)O | *Wisteria brachybotrys* | EBV and EV A71 | Inhibition of the attachment and entry of virus to host cell, reduces viral replication and protein synthesis | [Lalani et al. 2020] |
| C35/Cyanidin | 128861 | C1=CC(=C(C=C1C2=[O+]C3=CC(=CC(=C3C=C2O)O)O)O)O | Vitis vinifera and Aronia melanocarpa | West Nile virus, Zika virus, DENV and Influenza virus | Inhibition of the attachment, adsorption and entrance stages of virus particles | [Mohammadi Pour et al. 2019] |
| C36/Peonidin | 441773 | COC1=C(C=CC(=C1)C2=[O+]C3=CC(=CC(=C3C=C2O)O)O)O | Ribes nigrum,Vaccinium macrocarpon and Vitis vinifera | HSV-1, HSV-2, Hepatitis A virus and Influenza virus | Inhibit viral adhesion/adsorption and infectivity | [Mohammadi Pour et al. 2019] |
| C37/Malvidin | 159287 | COC1=CC(=CC(=C1O)OC)C2=[O+]C3=CC(=CC(=C3C=C2O)O)O | Ribes nigrum and Lycium barbarum | Coxsackievirus B1, Influenza A virus and Rotavirus | Inhibit the early phase of infection and replication of viruses | [Mohammadi Pour et al. 2019] |
| C38/Apigenidin | 159360 | C1=CC(=CC=C1C2=[O+]C3=CC(=CC(=C3C=C2)O)O)O.[Cl-] | *Prunus avium* and Vaccinium macrocarpon | Influenza virus | Inhibit the attachment and entry of viruses | [Mohammadi Pour et al. 2019]; [Lalani et al. 2020] |
| C39/Delphinidin | 68245 | C1=C(C=C(C(=C1O)O)O)C2=[O+]C3=CC(=CC(=C3C=C2O)O)O.[Cl-] | Phyllanthus phillyreifolius | West Nile virus, Zika virus and DENV | Direct effect on virus particles | [Mohammadi Pour et al. 2019] |
| C40/Eriodictyol | 440735 | C1C(OC2=CC(=CC(=C2C1=O)O)O)C3=CC(=C(C=C3)O)O | *Hordeum vulgare*, *Secale cereal* and Lyonia ovalifolia | HCV | Inhibition of viral multiplication | [Mohammadi Pour et al. 2019] |
| C41/Hesperetin | 72281 | COC1=C(C=C(C=C1)C2CC(=O)C3=C(C=C(C=C3O2)O)O)O | *Citrus aurantium*, *Citrus limon*, *Citrus reticulata* and *Mentha piperita* | Chikungunya virus, Yellow fever virus and HSV-1 | Inhibition of viral replication by interacting with viral nonstructural protein 2 and 3 | [Liu et al. 2009] |
| C42/Naringenin | 932 | C1C(OC2=CC(=CC(=C2C1=O)O)O)C3=CC=C(C=C3)O | *Citrus* paradise, *Citrus bergamia* and *Prunus cerasus* | HCV | block the assembly of intracellular  HCV particles | [Liu et al. 2009] |
| C43/Abyssinone II | 10064832 | CC(=CCC1=C(C=CC(=C1)C2CC(=O)C3=C(O2)C=C(C=C3)O)O)C | *Citrus reticulate* and *Prunus cerasus* | Influenza virus | Yet not detected | [Mohammadi Pour et al. 2019] |
| Control-1/  Hydroxychloroquine | 3652 | CCN(CCCC(C)NC1=C2C=CC(=CC2=NC=C1)Cl)CCO | Not applicable | SARS-CoV-2 | Not clearly known. Changes the pH of endosomes and believed to prevent viral entry, transport and post entry event | [Sing et al. 2020] |
| Control-2/Remdesivir | 121304016 | CCC(CC)COC(=O)C(C)NP(=O)(OCC1C(C(C(O1)(C#N)C2=CC=C3N2N=CN=C3N)O)O)OC4=CC=CC=C4 | Not applicable | SARS-CoV-2, SARS-CoV and MERS-CoV | Adenosine nucleotide analogue causes the inhibition of viral RNA polymerase | [Sing et al. 2020] |
| Control-3/  Camostat mesylate | 5284360 | CN(C)C(=O)COC(=O)CC1=CC=C(C=C1)OC(=O)C2=CC=C(C=C2)N=C(N)N.CS(=O)(=O)O | Not applicable | SARS-CoV-2 | Serine protease inhibitor causes blocking of viral maturation and entry into the host cell | [Sing et al. 2020] |

**Table S2.1**

| Target | Common name | Uniprot ID | ChEMBL ID | | Target Class | Probability* | Known actives (3D/2D) | |
| --- | --- | --- | --- | --- | --- | --- | --- | --- |
| Glyoxalase I | GLO1 | Q04760 | CHEMBL2424 | | Enzyme | 0.128898633299 | 0 / | 4 |
| Xanthine dehydrogenase | XDH | P47989 | CHEMBL1929 | | Oxidoreductase | 0.112748418065 | 0 / | 20 |
| Carbonic anhydrase II | CA2 | P00918 | CHEMBL205 | | Lyase | 0.112748418065 | 0 / | 11 |
| Carbonic anhydrase VII | CA7 | P43166 | CHEMBL2326 | | Lyase | 0.112748418065 | 0 / | 9 |
| Carbonic anhydrase XII | CA12 | O43570 | CHEMBL3242 | | Lyase | 0.112748418065 | 0 / | 12 |
| Carbonic anhydrase IV | CA4 | P22748 | CHEMBL3729 | | Lyase | 0.112748418065 | 0 / | 8 |
| Cytochrome P450 1B1 | CYP1B1 | Q16678 | CHEMBL4878 | | Cytochrome P450 | 0.112748418065 | 0 / | 43 |
| Thrombin | F2 | P00734 | CHEMBL204 | | Protease | 0.112748418065 | 2 / | 3 |
| Aldose reductase | AKR1B1 | P15121 | CHEMBL1900 | | Enzyme | 0.104671941128 | 0 / | 63 |
| ATP-binding cassette sub-family G member 2 | ABCG2 | Q9UNQ0 | CHEMBL5393 | | Primary active transporter | 0.104671941128 | 0 / | 44 |
| NADPH oxidase 4 | NOX4 | Q9NPH5 | CHEMBL1250375 | | Enzyme | 0.104671941128 | 0 / | 7 |
| LXR-alpha | NR1H3 | Q13133 | CHEMBL2808 | | Nuclear receptor | 0.104671941128 | 1 / | 1 |
| Lymphocyte differentiation antigen CD38 | CD38 | P28907 | CHEMBL4660 | | Enzyme | 0.104671941128 | 0 / | 3 |
| Adenosine A3 receptor | ADORA3 | P0DMS8 | CHEMBL256 | | Family A G protein- coupled receptor | 0.104671941128 | 0 / | 15 |
| P-glycoprotein 1 | ABCB1 | P08183 | CHEMBL4302 | | Primary active transporter | 0.104671941128 | 0 / | 45 |
| Microtubule- associated protein tau | MAPT | P10636 | CHEMBL1293224 | | Unclassified protein | 0.104671941128 | 0 / | 1 |
| Lysine-specific demethylase 4D-like | KDM4E | B2RXH2 | CHEMBL1293226 | | Eraser | 0.104671941128 | 0 / | 2 |
| G-protein coupled receptor 35 | GPR35 | Q9HC97 | CHEMBL1293267 | | Family A G protein- coupled receptor | 0.104671941128 | 0 / | 2 |
| Vasopressin V2 receptor | AVPR2 | P30518 | CHEMBL1790 | | Family A G protein- coupled receptor | 0.104671941128 | 0 / | 1 |
| DNA topoisomerase II alpha | TOP2A | P11388 | CHEMBL1806 | | Isomerase | 0.104671941128 | 0 / | 1 |
| Monoamine oxidase A | MAOA | P21397 | CHEMBL1951 | | Oxidoreductase | 0.104671941128 | 0 / | 3 |
| Insulin-like growth factor I receptor | IGF1R | P08069 | CHEMBL1957 | | Kinase | 0.104671941128 | 0 / | 2 |
| Tyrosine-protein kinase receptor FLT3 | FLT3 | P36888 | CHEMBL1974 | | Kinase | 0.104671941128 | 0 / | 6 |
| Cytochrome P450 19A1 | CYP19A1 | P11511 | CHEMBL1978 | | Cytochrome P450 | 0.104671941128 | 0 / | 12 |
| Insulin receptor | INSR | P06213 | CHEMBL1981 | | Kinase | 0.104671941128 | 0 / | 1 |
| Epidermal growth factor receptor erbB1 | EGFR | P00533 | CHEMBL203 | | Kinase | 0.104671941128 | 0 / | 23 |
| Target | Common name | Uniprot ID | ChEMBL ID | | Target Class | Probability* | Known actives (3D/2D) | |
| Serine/threonine- protein kinase PIM1 | PIM1 | P11309 | CHEMBL2147 | | Kinase | 0.104671941128 | 0 / | 4 |
| Arachidonate 5- lipoxygenase | ALOX5 | P09917 | CHEMBL215 | | Oxidoreductase | 0.104671941128 | 0 / | 48 |
| Serine/threonine- protein kinase Aurora-B | AURKB | Q96GD4 | CHEMBL2185 | | Kinase | 0.104671941128 | 0 / | 2 |
| Dopamine D4 receptor | DRD4 | P21917 | CHEMBL219 | | Family A G protein- coupled receptor | 0.104671941128 | 0 / | 1 |
| Acetylcholinesterase | ACHE | P22303 | CHEMBL220 | | Hydrolase | 0.104671941128 | 0 / | 29 |
| Adenosine A1 receptor (by homology) | ADORA1 | P30542 | CHEMBL226 | | Family A G protein- coupled receptor | 0.104671941128 | 0 / | 19 |
| Myosin light chain kinase, smooth muscle | MYLK | Q15746 | CHEMBL2428 | | Kinase | 0.104671941128 | 0 / | 1 |
| Myeloperoxidase | MPO | P05164 | CHEMBL2439 | | Enzyme | 0.104671941128 | 0 / | 1 |
| PI3-kinase p85-alpha subunit | PIK3R1 | P27986 | CHEMBL2506 | | Enzyme | 0.104671941128 | 0 / | 1 |
| Death-associated protein kinase 1 | DAPK1 | P53355 | CHEMBL2558 | | Kinase | 0.104671941128 | 0 / | 2 |
| Liver glycogen phosphorylase | PYGL | P06737 | CHEMBL2568 | | Enzyme | 0.104671941128 | 0 / | 1 |
| Tyrosine-protein kinase SYK | SYK | P43405 | CHEMBL2599 | | Kinase | 0.104671941128 | 0 / | 3 |
| Carbonic anhydrase I | CA1 | P00915 | CHEMBL261 | | Lyase | 0.104671941128 | 0 / | 5 |
| Glycogen synthase kinase-3 beta | GSK3B | P49841 | CHEMBL262 | | Kinase | 0.104671941128 | 0 / | 6 |
| Tyrosine-protein kinase SRC | SRC | P12931 | CHEMBL267 | | Kinase | 0.104671941128 | 0 / | 10 |
| Focal adhesion kinase 1 | PTK2 | Q05397 | CHEMBL2695 | | Kinase | 0.104671941128 | 0 / | 2 |
| Estradiol 17-beta- dehydrogenase 2 | HSD17B2 | P37059 | CHEMBL2789 | | Enzyme | 0.104671941128 | 0 / | 2 |
| Vascular endothelial growth factor receptor 2 | KDR | P35968 | CHEMBL279 | | Kinase | 0.104671941128 | 0 / | 2 |
| Matrix metalloproteinase 13 | MMP13 | P45452 | CHEMBL280 | | Protease | 0.104671941128 | 0 / | 1 |
| Matrix metalloproteinase 3 | MMP3 | P08254 | CHEMBL283 | | Protease | 0.104671941128 | 0 / | 1 |
| Carbonic anhydrase III | CA3 | P07451 | CHEMBL2885 | | Lyase | 0.104671941128 | 0 / | 1 |
| Arachidonate 15- lipoxygenase | ALOX15 | P16050 | CHEMBL2903 | | Enzyme | 0.104671941128 | 0 / | 4 |
| Multidrug resistance- associated protein 1 | ABCC1 | P33527 | CHEMBL3004 | | Primary active transporter | 0.104671941128 | 0 / | 11 |
| Serine/threonine- protein kinase PLK1 | PLK1 | P53350 | CHEMBL3024 | | Kinase | 0.104671941128 | 0 / | 2 |
| Carbonic anhydrase VI | CA6 | P23280 | CHEMBL3025 | | Lyase | 0.104671941128 | 0 / | 1 |
| Cyclin-dependent kinase 1 | CDK1 | P06493 | CHEMBL308 | | Kinase | 0.104671941128 | 0 / | 7 |
| Target Common Uniprot ChEMBL ID Target Class Probability* Known name ID actives  (3D/2D) | | | | | | | | |
| Matrix metalloproteinase 9 | MMP9 | P14780 | CHEMBL321 | | Protease | 0.104671941128 | 0 / | 2 |
| PI3-kinase p110- gamma subunit | PIK3CG | P48736 | CHEMBL3267 | | Enzyme | 0.104671941128 | 0 / | 1 |
| Matrix metalloproteinase 2 | MMP2 | P08253 | CHEMBL333 | | Protease | 0.104671941128 | 0 / | 2 |
| Protein kinase N1 | PKN1 | Q16512 | CHEMBL3384 | | Kinase | 0.104671941128 | 0 / | 2 |
| Carbonic anhydrase XIV | CA14 | Q9ULX7 | CHEMBL3510 | | Lyase | 0.104671941128 | 0 / | 1 |
| Carbonic anhydrase IX | CA9 | Q16790 | CHEMBL3594 | | Lyase | 0.104671941128 | 0 / | 5 |
| Casein kinase II alpha | CSNK2A1 | P68400 | CHEMBL3629 | | Kinase | 0.104671941128 | 0 / | 2 |
| Arachidonate 12- lipoxygenase | ALOX12 | P18054 | CHEMBL3687 | | Enzyme | 0.104671941128 | 0 / | 3 |
| Hepatocyte growth factor receptor | MET | P08581 | CHEMBL3717 | | Kinase | 0.104671941128 | 0 / | 3 |
| Serine/threonine- protein kinase NEK2 | NEK2 | P51955 | CHEMBL3835 | | Kinase | 0.104671941128 | 0 / | 2 |
| Interleukin-8 receptor A | CXCR1 | P25024 | CHEMBL4029 | | Family A G protein- coupled receptor | 0.104671941128 | 0 / | 1 |
| CaM kinase II beta | CAMK2B | Q13554 | CHEMBL4121 | | Kinase | 0.104671941128 | 0 / | 1 |
| ALK tyrosine kinase receptor | ALK | Q9UM73 | CHEMBL4247 | | Kinase | 0.104671941128 | 0 / | 2 |
| Serine/threonine- protein kinase AKT | AKT1 | P31749 | CHEMBL4282 | | Kinase | 0.104671941128 | 0 / | 4 |
| Serine/threonine- protein kinase NEK6 | NEK6 | Q9HC98 | CHEMBL4309 | | Kinase | 0.104671941128 | 0 / | 2 |
| Phospholipase A2 group 1B | PLA2G1B | P04054 | CHEMBL4426 | | Enzyme | 0.104671941128 | 0 / | 1 |
| Carbonic anhydrase VA | CA5A | P35218 | CHEMBL4789 | | Lyase | 0.104671941128 | 0 / | 1 |
| Beta-secretase 1 | BACE1 | P56817 | CHEMBL4822 | | Protease | 0.104671941128 | 0 / | 12 |
| Tyrosine-protein kinase receptor UFO | AXL | P30530 | CHEMBL4895 | | Kinase | 0.104671941128 | 0 / | 2 |
| DNA-(apurinic or apyrimidinic site) lyase | APEX1 | P27695 | CHEMBL5619 | | Enzyme | 0.104671941128 | 0 / | 1 |
| NUAK family SNF1- like kinase 1 | NUAK1 | O60285 | CHEMBL5784 | | Kinase | 0.104671941128 | 0 / | 2 |
| Aldo-keto reductase family 1 member C2 (by homology) | AKR1C2 | P52895 | CHEMBL5847 | | Enzyme | 0.104671941128 | 0 / | 1 |
| Aldo-keto reductase family 1 member C1 (by homology) | AKR1C1 | Q04828 | CHEMBL5905 | | Enzyme | 0.104671941128 | 0 / | 1 |
| Aldo-keto-reductase family 1 member C3 (by homology) | AKR1C3 | P42330 | CHEMBL4681 | | Enzyme | 0.104671941128 | 0 / | 1 |
| Aldo-keto reductase family 1 member C4 (by homology) | AKR1C4 | P17516 | CHEMBL4999 | | Enzyme | 0.104671941128 | 0 / | 1 |
| Target Common Uniprot ChEMBL ID Target Class Probability* Known name ID actives  (3D/2D) | | | | | | | | |
| Carbonic anhydrase XIII (by homology) | CA13 | Q8N1Q1 | | CHEMBL3912 | Lyase | 0.104671941128 | 0 / | 1 |
| Adenosine A2a  receptor (by  homology) | ADORA2A | P29274 | | CHEMBL251 | Family A G protein-  coupled receptor | 0.104671941128 | 0 / | 10 |
| Aldehyde reductase | AKR1A1 | P14550 | | CHEMBL2246 | Enzyme | 0.104671941128 | 0 / | 1 |
| Plasminogen | PLG | P00747 | | CHEMBL1801 | Protease | 0.104671941128 | 0 / | 3 |
| Cyclin-dependent  kinase 5/CDK5  activator 1 | CDK5R1  CDK5 | Q15078  Q00535 | | CHEMBL1907600 | Kinase | 0.104671941128 | 0 / | 5 |
| Cyclin-dependent  kinase 1/cyclin B | CCNB3  CDK1  CCNB1  CCNB2 | Q8WWL7  P06493  P14635  O95067 | | CHEMBL2094127 | Other cytosolic  protein | 0.104671941128 | 0 / | 4 |
| Cyclin-dependent  kinase 6 | CDK6 | Q00534 | | CHEMBL2508 | Kinase | 0.104671941128 | 0 / | 3 |
| Cyclin-dependent  kinase 2 | CDK2 | P24941 | | CHEMBL301 | Kinase | 0.104671941128 | 0 / | 8 |
| Arginase-1 (by  homology) | ARG1 | P05089 | | CHEMBL1075097 | Enzyme | 0.104671941128 | 0 / | 2 |
| Beta amyloid A4  protein | APP | P05067 | | CHEMBL2487 | Membrane  receptor | 0.104671941128 | 0 / | 10 |
| Delta opioid receptor | OPRD1 | P41143 | | CHEMBL236 | Family A G protein-  coupled receptor | 0.104671941128 | 0 / | 5 |
| Telomerase reverse  transcriptase | TERT | O14746 | | CHEMBL2916 | Enzyme | 0.104671941128 | 0 / | 18 |
| Beta-galactoside  alpha-2,6-  sialyltransferase 1 | ST6GAL1 | P15907 | | CHEMBL3596075 | Transferase | 0.104671941128 | 0 / | 2 |
| Phospholipase A2  group IIA | PLA2G2A | P14555 | | CHEMBL3474 | Enzyme | 0.104671941128 | 0 / | 2 |
| Tyrosinase | TYR | P14679 | | CHEMBL1973 | Oxidoreductase | 0.104671941128 | 0 / | 1 |
| Estradiol 17-beta-  dehydrogenase 1 | HSD17B1 | P14061 | | CHEMBL3181 | Enzyme | 0.104671941128 | 0 / | 3 |
| Aryl hydrocarbon  receptor | AHR | P35869 | | CHEMBL3201 | Transcription  factor | 0.104671941128 | 0 / | 1 |
| Estrogen-related  receptor alpha | ESRRA | P11474 | | CHEMBL3429 | Nuclear receptor | 0.104671941128 | 0 / | 1 |
| Cyclooxygenase-1 | PTGS1 | P23219 | | CHEMBL221 | Oxidoreductase | 0.104671941128 | 0 / | 3 |
| Phosphodiesterase  4B | PDE4B | Q07343 | | CHEMBL275 | Phosphodiesterase | 0.104671941128 | 0 / | 1 |
| Phosphodiesterase  4D | PDE4D | Q08499 | | CHEMBL288 | Phosphodiesterase | 0.104671941128 | 0 / | 5 |
| Induced myeloid  leukemia cell  differentiation  protein Mcl-1 | MCL1 | Q07820 | | CHEMBL4361 | Other cytosolic  protein | 0.104671941128 | 0 / | 1 |

**Table S2.2**

| Target | Common name | Uniprot ID | ChEMBL ID | Target Class | Probability* | Known actives (3D/2D) | |
| --- | --- | --- | --- | --- | --- | --- | --- |
| Lymphocyte differentiation antigen CD38 | CD38 | P28907 | CHEMBL4660 | Enzyme | 0.332035537736 | 2 / | 2 |
| Testis-specific androgen-binding protein | SHBG | P04278 | CHEMBL3305 | Secreted protein | 0.159189344372 | 0 / | 1 |
| NAD-dependent deacetylase sirtuin 1 | SIRT1 | Q96EB6 | CHEMBL4506 | Eraser | 0.127750341333 | 0 / | 1 |
| Cyclooxygenase-1 | PTGS1 | P23219 | CHEMBL221 | Oxidoreductase | 0.127750341333 | 0 / | 3 |
| Phosphodiesterase 4B | PDE4B | Q07343 | CHEMBL275 | Phosphodiesterase | 0.127750341333 | 0 / | 1 |
| Phosphodiesterase 4D | PDE4D | Q08499 | CHEMBL288 | Phosphodiesterase | 0.127750341333 | 0 / | 15 |
| Estrogen receptor alpha | ESR1 | P03372 | CHEMBL206 | Nuclear receptor | 0.119895126898 | 44 | / 35 |
| Estrogen receptor beta | ESR2 | Q92731 | CHEMBL242 | Nuclear receptor | 0.119895126898 | 49 | / 35 |
| Androgen Receptor | AR | P10275 | CHEMBL1871 | Nuclear receptor | 0.119895126898 | 0 / | 16 |
| LXR-alpha | NR1H3 | Q13133 | CHEMBL2808 | Nuclear receptor | 0.119895126898 | 0 / | 1 |
| Aldose reductase (by homology) | AKR1B1 | P15121 | CHEMBL1900 | Enzyme | 0.119895126898 | 6 / | 58 |
| AMY1C | AMY1A | P04745 | CHEMBL2478 | Enzyme | 0.119895126898 | 0 / | 1 |
| G protein-coupled receptor kinase 6 | GRK6 | P43250 | CHEMBL6144 | Kinase | 0.119895126898 | 0 / | 4 |
| Tankyrase-2 | TNKS2 | Q9H2K2 | CHEMBL6154 | Enzyme | 0.119895126898 | 4 / | 12 |
| Tankyrase-1 | TNKS | O95271 | CHEMBL6164 | Enzyme | 0.119895126898 | 4 / | 28 |
| Monoamine oxidase A | MAOA | P21397 | CHEMBL1951 | Oxidoreductase | 0.112041901328 | 5 / | 6 |
| Adenosine A1 receptor (by homology) | ADORA1 | P30542 | CHEMBL226 | Family A G protein- coupled receptor | 0.112041901328 | 4 / | 22 |
| Adenosine A2a receptor | ADORA2A | P29274 | CHEMBL251 | Family A G protein- coupled receptor | 0.112041901328 | 3 / | 11 |
| Estradiol 17-beta- dehydrogenase 1 | HSD17B1 | P14061 | CHEMBL3181 | Enzyme | 0.112041901328 | 4 / | 4 |
| ATP-binding cassette sub-family G member 2 | ABCG2 | Q9UNQ0 | CHEMBL5393 | Primary active transporter | 0.112041901328 | 5 / | 46 |
| Cytochrome P450 19A1 | CYP19A1 | P11511 | CHEMBL1978 | Cytochrome P450 | 0.112041901328 | 3 / | 13 |
| NADPH oxidase 4 | NOX4 | Q9NPH5 | CHEMBL1250375 | Enzyme | 0.112041901328 | 3 / | 6 |
| Cyclin-dependent kinase 5/CDK5 activator 1 | CDK5R1 CDK5 | Q15078 Q00535 | CHEMBL1907600 | Kinase | 0.112041901328 | 5 / | 12 |
| Xanthine dehydrogenase | XDH | P47989 | CHEMBL1929 | Oxidoreductase | 0.112041901328 | 8 / | 18 |
| Tyrosine-protein kinase receptor FLT3 | FLT3 | P36888 | CHEMBL1974 | Kinase | 0.112041901328 | 3 / | 6 |
| Cyclin-dependent kinase 1/cyclin B | CCNB3 CDK1 CCNB1 CCNB2 | P06493 P14635 O95067  Q8WWL7 | CHEMBL2094127 | Other cytosolic protein | 0.112041901328 | 4 / | 4 |
| Target | Common name | Uniprot ID | ChEMBL ID | Target Class | Probability* | Known actives (3D/2D) | |
| Acetylcholinesterase | ACHE | P22303 | CHEMBL220 | Hydrolase | 0.112041901328 | 3 / 23 | |
| Cyclooxygenase-2 | PTGS2 | P35354 | CHEMBL230 | Oxidoreductase | 0.112041901328 | 1 / 7 | |
| Cyclin-dependent kinase 6 | CDK6 | Q00534 | CHEMBL2508 | Kinase | 0.112041901328 | 3 / 3 | |
| Tyrosine-protein kinase SYK | SYK | P43405 | CHEMBL2599 | Kinase | 0.112041901328 | 2 / 3 | |
| Glycogen synthase kinase-3 beta | GSK3B | P49841 | CHEMBL262 | Kinase | 0.112041901328 | 2 / 13 | |
| Multidrug resistance- associated protein 1 | ABCC1 | P33527 | CHEMBL3004 | Primary active transporter | 0.112041901328 | 5 / 10 | |
| Transthyretin | TTR | P02766 | CHEMBL3194 | Secreted protein | 0.112041901328 | 2 / 2 | |
| Casein kinase II alpha | CSNK2A1 | P68400 | CHEMBL3629 | Kinase | 0.112041901328 | 2 / 2 | |
| Cystic fibrosis transmembrane conductance regulator | CFTR | P13569 | CHEMBL4051 | Other ion channel | 0.112041901328 | 1 / 1 | |
| Cytochrome P450 1B1 | CYP1B1 | Q16678 | CHEMBL4878 | Cytochrome P450 | 0.112041901328 | 10 / 40 | |
| Aldo-keto reductase family 1 member B10 | AKR1B10 | O60218 | CHEMBL5983 | Enzyme | 0.112041901328 | 2 / | 3 |
| Thrombin | F2 | P00734 | CHEMBL204 | Protease | 0.112041901328 | 8 / | 3 |
| Lysine-specific demethylase 4D-like | KDM4E | B2RXH2 | CHEMBL1293226 | Eraser | 0.112041901328 | 0 / | 2 |
| Carbonic anhydrase VII | CA7 | P43166 | CHEMBL2326 | Lyase | 0.112041901328 | 6 / | 8 |
| Carbonic anhydrase XII | CA12 | O43570 | CHEMBL3242 | Lyase | 0.112041901328 | 6 / | 12 |
| Carbonic anhydrase IV | CA4 | P22748 | CHEMBL3729 | Lyase | 0.112041901328 | 5 / | 7 |
| Receptor-type tyrosine-protein phosphatase S | PTPRS | Q13332 | CHEMBL2396508 | Phosphatase | 0.112041901328 | 1 / | 8 |
| Arachidonate 5- lipoxygenase | ALOX5 | P09917 | CHEMBL215 | Oxidoreductase | 0.112041901328 | 3 / | 35 |
| Poly [ADP-ribose] polymerase-1 | PARP1 | P09874 | CHEMBL3105 | Enzyme | 0.112041901328 | 3 / | 9 |
| Glyoxalase I | GLO1 | Q04760 | CHEMBL2424 | Enzyme | 0.112041901328 | 1 / | 4 |
| NEDD8-activating enzyme E1 regulatory subunit | NAE1 | Q13564 | CHEMBL2016431 | Unclassified protein | 0.112041901328 | 0 / | 1 |
| P-glycoprotein 1 | ABCB1 | P08183 | CHEMBL4302 | Primary active transporter | 0.112041901328 | 8 / | 41 |
| Carbonic anhydrase II | CA2 | P00918 | CHEMBL205 | Lyase | 0.112041901328 | 5 / | 9 |
| Stem cell growth factor receptor | KIT | P10721 | CHEMBL1936 | Kinase | 0.112041901328 | 0 / | 2 |
| Delta opioid receptor | OPRD1 | P41143 | CHEMBL236 | Family A G protein- coupled receptor | 0.112041901328 | 0 / | 5 |
| Target Common Uniprot ChEMBL ID Target Class Probability* Known name ID actives  (3D/2D) | | | | | | | |
| Monoamine oxidase B | MAOB | P27338 | CHEMBL2039 | Oxidoreductase | 0.112041901328 | 0 / | 13 |
| Carbonic anhydrase I | CA1 | P00915 | CHEMBL261 | Lyase | 0.112041901328 | 1 / | 6 |
| Carbonic anhydrase IX | CA9 | Q16790 | CHEMBL3594 | Lyase | 0.112041901328 | 1 / | 7 |
| Carbonyl reductase [NADPH] 1 | CBR1 | P16152 | CHEMBL5586 | Enzyme | 0.112041901328 | 1 / | 2 |
| Arginase-1 (by homology) | ARG1 | P05089 | CHEMBL1075097 | Enzyme | 0.112041901328 | 2 / | 2 |
| Prostaglandin E synthase | PTGES | O14684 | CHEMBL5658 | Enzyme | 0.112041901328 | 0 / | 2 |
| Tyrosinase | TYR | P14679 | CHEMBL1973 | Oxidoreductase | 0.112041901328 | 2 / | 2 |
| Beta-galactoside alpha-2,6- sialyltransferase 1 | ST6GAL1 | P15907 | CHEMBL3596075 | Transferase | 0.112041901328 | 0 / | 2 |
| Arachidonate 15- lipoxygenase | ALOX15 | P16050 | CHEMBL2903 | Enzyme | 0.112041901328 | 1 / | 5 |
| Arachidonate 12- lipoxygenase | ALOX12 | P18054 | CHEMBL3687 | Enzyme | 0.112041901328 | 2 / | 6 |
| Serine/threonine- protein kinase PIM1 | PIM1 | P11309 | CHEMBL2147 | Kinase | 0.112041901328 | 3 / | 6 |
| Beta amyloid A4 protein | APP | P05067 | CHEMBL2487 | Membrane receptor | 0.112041901328 | 1 / | 14 |
| Matrix metalloproteinase 9 | MMP9 | P14780 | CHEMBL321 | Protease | 0.112041901328 | 1 / | 2 |
| Matrix metalloproteinase 2 | MMP2 | P08253 | CHEMBL333 | Protease | 0.112041901328 | 1 / | 2 |
| Matrix metalloproteinase 12 | MMP12 | P39900 | CHEMBL4393 | Protease | 0.112041901328 | 1 / | 2 |
| DNA topoisomerase I (by homology) | TOP1 | P11387 | CHEMBL1781 | Isomerase | 0.112041901328 | 1 / | 1 |
| Estrogen-related receptor alpha | ESRRA | P11474 | CHEMBL3429 | Nuclear receptor | 0.112041901328 | 2 / | 2 |
| Inhibitor of nuclear factor kappa B kinase beta subunit | IKBKB | O14920 | CHEMBL1991 | Kinase | 0.112041901328 | 0 / | 1 |
| Neurotrophic tyrosine kinase receptor type 2 | NTRK2 | Q16620 | CHEMBL4898 | Kinase | 0.112041901328 | 0 / | 1 |
| G-protein coupled receptor 35 | GPR35 | Q9HC97 | CHEMBL1293267 | Family A G protein- coupled receptor | 0.112041901328 | 0 / | 2 |
| Death-associated protein kinase 1 | DAPK1 | P53355 | CHEMBL2558 | Kinase | 0.112041901328 | 0 / | 2 |
| DNA-3-  methyladenine glycosylase | MPG | P29372 | CHEMBL3396943 | Enzyme | 0.112041901328 | 0 / | 1 |
| Solute carrier family 22 member 12 | SLC22A12 | Q96S37 | CHEMBL6120 | Electrochemical transporter | 0.112041901328 | 0 / | 1 |
| Calmodulin | CALM1 | P62158 | CHEMBL6093 | Unclassified protein | 0.112041901328 | 0 / | 1 |
| Cyclin-dependent kinase 1 | CDK1 | P06493 | CHEMBL308 | Kinase | 0.112041901328 | 1 / | 8 |
| Target Common Uniprot ChEMBL ID Target Class Probability* Known name ID actives  (3D/2D) | | | | | | | |
| Microtubule- associated protein tau | MAPT | P10636 | CHEMBL1293224 | Unclassified protein | 0.112041901328 | 0 / | 1 |
| Vasopressin V2 receptor | AVPR2 | P30518 | CHEMBL1790 | Family A G protein- coupled receptor | 0.112041901328 | 0 / | 1 |
| DNA topoisomerase II alpha | TOP2A | P11388 | CHEMBL1806 | Isomerase | 0.112041901328 | 0 / | 1 |
| Insulin receptor | INSR | P06213 | CHEMBL1981 | Kinase | 0.112041901328 | 0 / | 1 |
| Dopamine D4 receptor | DRD4 | P21917 | CHEMBL219 | Family A G protein- coupled receptor | 0.112041901328 | 0 / | 1 |
| Myosin light chain kinase, smooth muscle | MYLK | Q15746 | CHEMBL2428 | Kinase | 0.112041901328 | 0 / | 1 |
| Myeloperoxidase | MPO | P05164 | CHEMBL2439 | Enzyme | 0.112041901328 | 0 / | 1 |
| PI3-kinase p85-alpha subunit | PIK3R1 | P27986 | CHEMBL2506 | Enzyme | 0.112041901328 | 0 / | 1 |
| Liver glycogen phosphorylase | PYGL | P06737 | CHEMBL2568 | Enzyme | 0.112041901328 | 0 / | 1 |
| Tyrosine-protein kinase SRC | SRC | P12931 | CHEMBL267 | Kinase | 0.112041901328 | 0 / | 2 |
| Focal adhesion kinase 1 | PTK2 | Q05397 | CHEMBL2695 | Kinase | 0.112041901328 | 0 / | 2 |
| Matrix metalloproteinase 13 | MMP13 | P45452 | CHEMBL280 | Protease | 0.112041901328 | 0 / | 1 |
| Matrix metalloproteinase 3 | MMP3 | P08254 | CHEMBL283 | Protease | 0.112041901328 | 0 / | 1 |
| Carbonic anhydrase III | CA3 | P07451 | CHEMBL2885 | Lyase | 0.112041901328 | 0 / | 1 |
| Carbonic anhydrase VI | CA6 | P23280 | CHEMBL3025 | Lyase | 0.112041901328 | 0 / | 1 |
| PI3-kinase p110- gamma subunit | PIK3CG | P48736 | CHEMBL3267 | Enzyme | 0.112041901328 | 0 / | 1 |
| Protein-tyrosine phosphatase 1B | PTPN1 | P18031 | CHEMBL335 | Phosphatase | 0.112041901328 | 0 / | 24 |
| Protein kinase N1 | PKN1 | Q16512 | CHEMBL3384 | Kinase | 0.112041901328 | 0 / | 2 |
| Carbonic anhydrase XIV | CA14 | Q9ULX7 | CHEMBL3510 | Lyase | 0.112041901328 | 0 / | 1 |
| Serine/threonine- protein kinase NEK2 | NEK2 | P51955 | CHEMBL3835 | Kinase | 0.112041901328 | 0 / | 2 |
| Interleukin-8 receptor A | CXCR1 | P25024 | CHEMBL4029 | Family A G protein- coupled receptor | 0.112041901328 | 0 / | 1 |
| CaM kinase II beta | CAMK2B | Q13554 | CHEMBL4121 | Kinase | 0.112041901328 | 0 / | 1 |
| Serine/threonine- protein kinase AKT | AKT1 | P31749 | CHEMBL4282 | Kinase | 0.112041901328 | 0 / | 4 |
| Serine/threonine- protein kinase NEK6 | NEK6 | Q9HC98 | CHEMBL4309 | Kinase | 0.112041901328 | 0 / | 2 |

**References**

Andres A, Donovan S, Kuhlenschmidt M (2009) Soy isoflavones and virus infections. J Nutr Biochem 20(8):563-569

Demeule M, Michaud-Levesque J, Annabi B et al. (2002) Green tea catechins as novel antitumor and antiangiogenic compounds. Curr Med Chem Anticancer Agents 2:441–463

Evers DL, Chao CF, Wang X et al. (2005) Human cytomegalovirus-inhibitory flavonoids: studies on antiviral activity and mechanism of action. Antiviral Res 68:124–134

Fritz D, Venturi C, Cargnin S, Schripsema J, Roehe P, Montanha J et al. (2007) Herpes virus inhibitory substances from *Hypericum connatum* Lam., a plant used in southern Brazil to treat oral lesions. J Ethnopharmacol 113(3):517-520

He M, Min J, Kong W, He X, Li J, Peng B (2016) A review on the pharmacological effects of vitexin and isovitexin. Fitoterapia 115:74-85

Jou S, Chen C, Guh J, Lee C, Lee S (2004) Flavonol Glycosides and Cytotoxic Triterpenoids from *Alphitonia philippinensis*. J Chin Chem Soc 51(4):827-834

Lalani S, Poh CL (2020) Flavonoids as Antiviral Agents for *Enterovirus* A71 (EV-A71). Viruses 12(2):184

Li J, Song D, Wang S, Dai Y, Zhou J, Gu J (2020) Antiviral Effect of Epigallocatechin Gallate via Impairing Porcine Circovirus Type 2 Attachment to Host Cell Receptor. Viruses 12(2):176

Lin Y, Flavin M, Schure R, Chen F, Sidwell R, Barnard D et al. (1999) Antiviral Activities of Biflavonoids. Planta Med 65(2):120-125

Liu A, Shu S, Qin H, Lee S, Wang Y, Du G (2009) In vitroAnti-Influenza Viral Activities of Constituents from *Caesalpinia sappan*. Planta Med 75(04):337-339

Malhotra B, Onyilagha J, Bohm B, Towers G, James D, Harborne J et al. (1996) Inhibition of tomato ringspot virus by flavonoids. Phytochemistry 43(6):1271-1276

McKee D, Sternberg A, Stange U, Laufer S, Naujokat C (2020) Candidate drugs against SARS-CoV-2 and COVID-19. Pharmacol Res 157:104859

Mohammadi Pour P, Fakhri S, Asgary S, Farzaei M, Echeverría J (2019) The Signaling Pathways, and Therapeutic Targets of Antiviral Agents: Focusing on the Antiviral Approaches and Clinical Perspectives of Anthocyanins in the Management of Viral Diseases. Front Pharmacol 10:1207

Nagai E, Iwai M, Koketsu R et al. (2019) Anti-Influenza Virus Activity of Adlay Tea Components. Plant Foods Hum Nutr 74(4):538-543

Nothias-Scaglia L, Retailleau P, Paolini J, Pannecouque C, Neyts J, Dumontet V et al. (2014) Jatrophane Diterpenes as Inhibitors of Chikungunya Virus Replication: Structure–Activity Relationship and Discovery of a Potent Lead. J Nat Prod 77(6):1505-1512

Semple S, Nobbs S, Pyke S, Reynolds G, Flower R (1999) Antiviral flavonoid from Pterocaulon sphacelatum, an Australian Aboriginal medicine. J Ethnopharmacol 68(1-3):283-288

Seong R, Kim J, Shin O (2018) Wogonin, a flavonoid isolated from *Scutellaria baicalensis*, has anti-viral activities against influenza infection via modulation of AMPK pathways. Acta Virol 62(01):78-85

Singh A, Singh A, Shaikh A, Singh R, Misra A (2020) Chloroquine and hydroxychloroquine in the treatment of COVID-19 with or without diabetes: A systematic search and a narrative review with a special reference to India and other developing countries. Diabetes & Metabolic Syndrome: Clinical Research & Reviews 14(3):241-246

Song J, Kwon B, Jang H, Kang H, Cho S, Park K et al. (2015) Antiviral Activity of Chrysin Derivatives against Coxsackievirus B3 in vitro and in vivo. Biomol Ther 23(5):465-470

Wang M, Firrman J, Liu L, Yam K (2019) A Review on Flavonoid Apigenin: Dietary Intake, ADME, Antimicrobial Effects, and Interactions with Human Gut Microbiota. BioMed Res Int 2019:1-18

Wu M, Zhang Q, Yi D, Wu T, Chen H, Guo S et al. (2020) Quantitative Proteomic Analysis Reveals Antiviral and Anti-inflammatory Effects of Puerarin in Piglets Infected With Porcine Epidemic Diarrhea Virus. Front Immunol 11:169

Xu L, Jiang W, Jia H, Zheng L, Xing J, Liu A et al. (2020) Discovery of Multitarget-Directed Ligands Against Influenza A Virus From Compound Yizhihao Through a Predictive System for Compound-Protein Interactions. Front Cell Infect Microbiol 10:16

Yang F, Zhou W, Liu A, Qin H, Lee S, Wang Y et al. (2012) The Protective Effect of 3-Deoxysappanchalcone on In Vitro Influenza Virus-induced Apoptosis and Inflammation. Planta Medica 78(10):968-973

Yu C, Zhang P, Lou L, Wang Y (2019) Perspectives Regarding the Role of Biochanin A in Humans. Front Pharmacol 10:793

Yu Y, Miyashiro H, Nakamura N, Hattori M, Park J (2007) Effects of triterpenoids and flavonoids isolated from alnus firma on HIV-1 viral enzymes. Arch Pharm Res 30(7):820-826

Yu Y, Li Z, Guo R, Qian J, Zhang H, Zhang J et al. (2019) Ononin, sec-O-β-d-glucosylhamaudol and astragaloside I: antiviral lead compounds identified via high throughput screening and biological validation from traditional Chinese medicine Zhongjing formulary. Pharmacol Res 145:104248

Zakaryan H, Arabyan E, Oo A, Zandi K (2017) Flavonoids: promising natural compounds against viral infections. Arch Virol 162(9):2539-2551
